# Anoxia-Reoxygenation Regulates Mitochondrial Dynamics through the Hypoxia Response Pathway, SKN-1/Nrf, and Stomatin-Like Protein STL-1/SLP-2

**DOI:** 10.1371/journal.pgen.1004063

**Published:** 2013-12-26

**Authors:** Piya Ghose, Eun Chan Park, Alexandra Tabakin, Nathaly Salazar-Vasquez, Christopher Rongo

**Affiliations:** 1 The Waksman Institute, Department of Genetics, Rutgers The State University of New Jersey, Piscataway, New Jersey, United States of America; 2 The Graduate Program in Neuroscience, Rutgers The State University of New Jersey, Piscataway, New Jersey, United States of America; 3 The Graduate Program in Genetics and Microbiology, Rutgers The State University of New Jersey, Piscataway, New Jersey, United States of America; The University of Texas Health Science Center at Houston, United States of America

## Abstract

Many aerobic organisms encounter oxygen-deprived environments and thus must have adaptive mechanisms to survive such stress. It is important to understand how mitochondria respond to oxygen deprivation given the critical role they play in using oxygen to generate cellular energy. Here we examine mitochondrial stress response in *C. elegans*, which adapt to extreme oxygen deprivation (anoxia, less than 0.1% oxygen) by entering into a reversible suspended animation state of locomotory arrest. We show that neuronal mitochondria undergo DRP-1-dependent fission in response to anoxia and undergo refusion upon reoxygenation. The hypoxia response pathway, including EGL-9 and HIF-1, is not required for anoxia-induced fission, but does regulate mitochondrial reconstitution during reoxygenation. Mutants for *egl-9* exhibit a rapid refusion of mitochondria and a rapid behavioral recovery from suspended animation during reoxygenation; both phenotypes require HIF-1. Mitochondria are significantly larger in *egl-9* mutants after reoxygenation, a phenotype similar to stress-induced mitochondria hyperfusion (SIMH). Anoxia results in mitochondrial oxidative stress, and the oxidative response factor SKN-1/Nrf is required for both rapid mitochondrial refusion and rapid behavioral recovery during reoxygenation. In response to anoxia, SKN-1 promotes the expression of the mitochondrial resident protein Stomatin-like 1 (STL-1), which helps facilitate mitochondrial dynamics following anoxia. Our results suggest the existence of a conserved anoxic stress response involving changes in mitochondrial fission and fusion.

## Author Summary

Oxygen deprivation plays a role in multiple human diseases ranging from heart attack, ischemic stroke, and traumatic injury. Aerobic organisms use oxygen to generate cellular energy in mitochondria; thus, oxygen deprivation results in energy depletion. Low oxygen can be catastrophic in tissues like the nervous system, which has high-energy demands and few glycolytic reserves. By contrast, other cells, including stem cells and cancerous cells within tumors, adapt and thrive in low oxygen. We are just beginning to understand how different organisms and even different cell types within the same organism respond to low oxygen conditions. The response of mitochondria to oxygen deprivation is particularly critical given their role in aerobic energy production. In addition, mitochondria actively injure cells during oxygen deprivation through the generation of reactive oxygen species, the disruption of calcium homeostasis, and the activation of cell death pathways. Here we use a genetic approach to show that mitochondria undergo fission during oxygen deprivation and refusion upon oxygen restoration. The hypoxia response pathway and the oxidative stress response pathway together modulate this response. We identify a new factor, stomatin-like protein, as a promoter of mitochondrial fusion in response to oxygen deprivation stress. Our findings uncover a new mechanism – regulated mitochondrial dynamics – by which cells adapt to oxygen deprivation stress.

## Introduction

Oxygen is critical for the survival of obligate aerobic organisms, and such organisms have evolved stress responses to avoid or offset damage when confronted with low oxygen (hypoxia) or no oxygen (anoxia) in their natural environment. Indeed, many species have evolved mechanisms that allow them to adapt to oxygen deprivation and its consequences for prolonged periods [1], [2], [3], [4]. Yet even within the same species, adaptation to oxygen deprivation varies dramatically according to tissue type, age, and sex [5], [6], [7], [8], [9]. Within humans, oxygen deprivation can be a normal physiological condition; for example, a low oxygen microenvironment is required for stem cells to maintain their undifferentiated state [10]. Oxygen deprivation is also a critical facet of multiple human pathologies. Cancerous cells have been observed to adapt to the hypoxic microenvironment of solid tumors, and this adaptive response can promote malignancy [11]. Oxygen deprivation also has devastating consequences during ischemic stroke and heart attack [12], [13], [14]. A better understanding of the responses to oxygen deprivation stress should therefore lend itself to the development of new therapies for treating diseases ranging from ischemic damage to cancer.

Oxygen deprivation has major consequences for mitochondria in particular given their role as the hub for aerobic metabolism and ATP generation. Mitochondria play an active role in cellular injury during oxygen deprivation (e.g., during ischemia and reperfusion) through the generation of reactive oxygen species (ROS), the disruption of calcium homeostasis, and the activation of cell death signaling pathways [15], [16], [17]. Given the potential threat from mitochondria during oxygen deprivation, cells execute stress responses that include changes in mitochondrial biogenesis and removal [18]. Mitochondria exist in a dynamic state of fission and fusion, and this balance can be tipped by stress or disease towards fission and subsequent autophagy/mitophagy of the fragmented mitochondrial products [19]. Mitochondria sometimes respond to stress by showing increased levels of fusion, including a form of augmented fusion mediated by the prohibitin-like SLP-2 protein, termed stress-induced mitochondrial hyperfusion (SIMH) [20]. While the exact purpose for these changes in morphological dynamics is unclear, regulated mitochondrial dynamics appear to be particularly important in neurons, which have high energy demands but little in the way of glycolytic reserves. Correspondingly, altered mitochondrial dynamics are observed in multiple forms of neurodegeneration, which is particularly sensitive to mitochondrial function [21], [22].

*C. elegans* is used as a model for oxygen deprivation stress response. Soil nematodes naturally encounter environmental conditions with variable oxygen concentrations [23], [24]. Under conditions of hypoxia (∼1% oxygen), *C. elegans* decreases its oxygen consumption and alters its locomotion and aerotaxis behaviors, but continues to develop and reproduce [25], [26], [27], [28], [29], [30]. Under conditions of anoxia (<0.1% oxygen), *C. elegans* undergoes developmental arrest and enters a state of suspended animation, eventually dying after long term exposure [24], [31], [32], [33], [34].

*C. elegans* also exhibits different signaling responses to anoxia and hypoxia. For example, in response to hypoxia *C. elegans* employs the conserved hypoxia response pathway. When oxygen levels are sufficiently high, the prolyl hydroxylase EGL-9 uses molecular oxygen, 2-oxoglutarate, and iron to hydroxylate key proline side chains on HIF-1, the hypoxia-inducible factor, resulting in HIF-1 protein degradation [25], [35], [36], [37], [38]. Under hypoxic conditions, HIF-1 remains stable, acting as a transcription factor to regulate gene expression [39], [40]. The signaling response pathway to anoxia is less well understood, but mutations in the insulin/IGF signaling pathway, the AMP Kinase (AMPK) pathway, and the p38 MAP Kinase (MAPK) pathway can sensitize *C. elegans* to anoxia-induced death [32], [41], [42], [43]. These signaling molecules are implicated in responding to oxidative stress and ROS, suggesting that ROS and alterations in mitochondria might contribute to anoxia-induced damage, although the exact cellular and molecular mechanisms remain unclear.

Here we use cell biological and genetic approaches to address how *C. elegans* mitochondria respond to anoxia. We show that anoxia induces mitochondrial fission in *C. elegans* neurons, whereas reoxygenation results in mitochondrial refusion. We show that the hypoxia response pathway and the oxidative stress response factor SKN-1/Nrf promote mitochondrial refusion during reoxygenation. Specifically, we find that mitochondria undergo hyperfusion when EGL-9 activity is absent, an effect that is similar to that of SIMH in stressed mammalian cells. We show that SKN-1/Nrf promotes the expression of STL-1, the *C. elegans* ortholog of SLP-2, a key mediator of mammalian SIMH, and that both SKN-1/Nrf and STL-1 are essential for the hyperfusion observed during reoxygenation following anoxia. Our results suggest that anoxia regulates mitochondrial dynamics through the hypoxia response pathway and the oxidative stress pathway by promoting hyperfusion during recovery, and that the mitochondrial resident protein STL-1 is required for this specific stress response.

## Results

### Anoxia promotes suspended animation and eventually death in *C. elegans*

**Figure 1:**
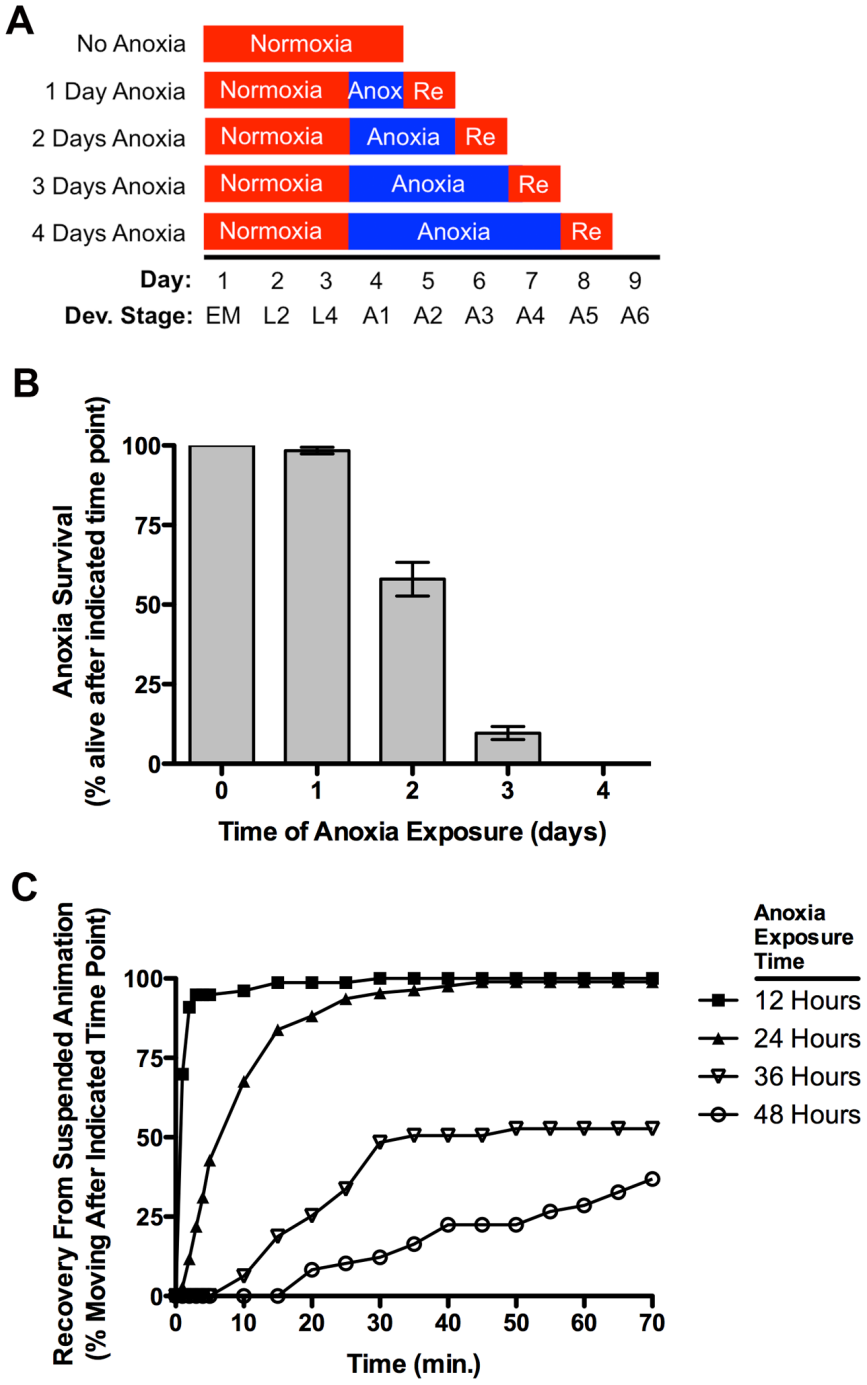
Anoxia promotes suspended animation and eventually death in *C. elegans*. (A) Protocol for *C. elegans* anoxia treatment. The x-axis indicates time (in days since fertilization) and developmental stage (“EM” for embryo, “L2” and “L4” for respective larval stages, and “A1–A6” for the indicated day of mature adulthood). Boxes indicate the treatment during that particular period, with red indicating exposure to a normoxic environment (or the 1-day reoxygenation, labeled as “Re”) and blue indicating exposure to an anoxic environment. (B) Mean percentage of animals surviving after the given exposure time to anoxia. Error bars indicate SEM. (C) Mean percentage of animals moving (i.e., recovered from suspended animation) at the given time point following reoxygenation (post-anoxia). Individually plotted lines represent recovery following 12 (filled squares), 24 (filled triangles), 36 (empty triangles), and 48 (empty circles) hours of anoxia exposure. N = 15–35 animals per condition and/or genotype.

To examine the effects of anoxia on adult *C. elegans*, we raised wild-type embryos and larvae under normoxic conditions and then shifted developmentally synchronized animals (24 hours following the L4 larval molt) to anaerobic biobags (anoxia) at 20°C for various lengths of time (Figure 1A). We then returned the animals back to normoxic conditions (reoxygenation) and allowed them to recover for 24 hours. Greater than 95% of wild-type animals survived 24 hours of short-term anoxia; however, about half survived 48 hours of long-term anoxic exposure, and fewer than 10% survived 72 hours of anoxia (Figure 1B), rates similar to those previously reported for solid agar culturing [32], [33], [44]. Upon anoxic exposure, animals entered into a previously described state of suspended animation in which they did not move [24]. Suspended animation was rapidly reversible upon reoxygenation, with nematodes emerging from suspension at a rate that reflected the duration of the initial anoxic exposure (Figure 1C).

### Anoxia promotes DRP-1-dependent mitochondrial fission

**Figure 2:**
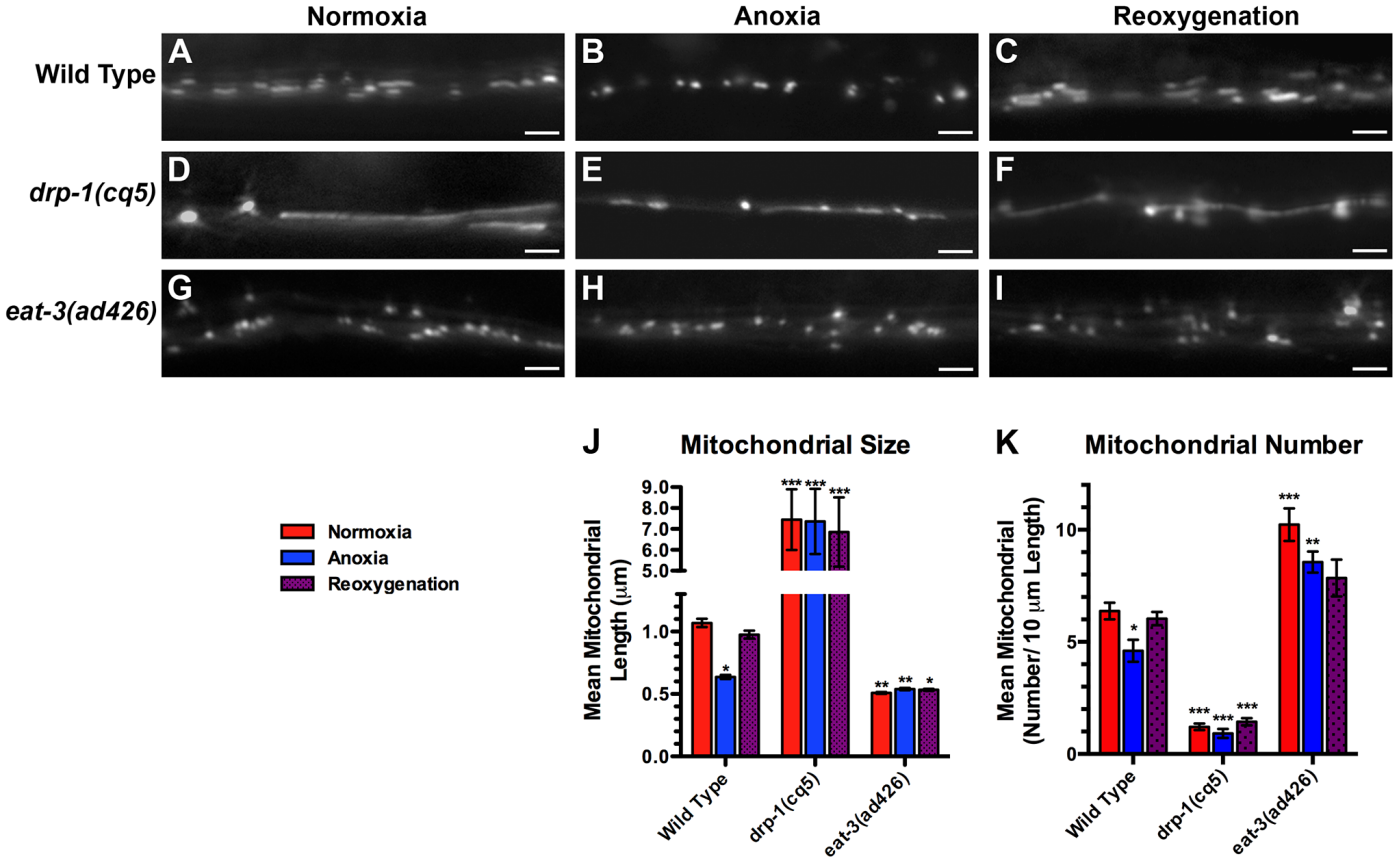
Anoxia promotes DRP-1-dependent mitochondrial fission. The fluorescence of MitoGFP was observed along ventral cord neurites of (A,B,C) wild-type animals, (D,E,F) *drp-1(cq5)* mutants, and (G,H,I) *eat-3(ad426)* mutants under conditions of (A,D,G) normoxia, (B,E,H) following 24 hours of anoxia, or (C,F,I) following 3 hours of reoxygenation post-anoxia. (J,K) Quantification of the mean (J) length and (K) number of mitochondria along the ventral cord for the indicated genotypes and conditions. Red bars indicate normoxia, blue bars indicate anoxia, and purple stippled bars indicate reoxygenation. ANOVA followed by Dunnett's multiple comparison to wild type, normoxia (*p<0.05, **p<0.01, ***p<0.001). N = 15–35 animals per condition and/or genotype. Error bars indicate SEM. Bar, 5 µm.

We sought to understand the cell biological mechanism behind anoxia-induced suspended animation in *C. elegans*. Given the sensitivity of neurons to oxygen levels, we focused our attention on neurons. We also considered the critical role mitochondria play in both neuronal function and oxygen consumption, and we proceeded to test specifically whether mitochondria in *C. elegans* neurons respond to changes in ambient oxygen. To visualize neuronal mitochondria, we generated transgenic animals expressing a mitochondrial matrix-directed leader sequence attached to GFP (MitoGFP) in the command interneurons using the *glr-1* promoter. We obtained two independently integrated transgenic lines, called *odIs70* and *odIs71*, which showed no apparent differences other than chromosomal position. Fluorescently labeled mitochondria from these lines appeared as elongated structures and smaller circular puncta along the ventral cord neurites of these neurons, and they showed a more reticulate orientation in the neuronal cell bodies (Figure 2A and data not shown).

Various cellular stresses can induce changes in mitochondrial dynamics [19], [20], [21], [45]. We tested whether the stress of anoxia exposure could regulate mitochondrial dynamics in *C. elegans* neurons expressing MitoGFP. We found that 24 hours of anoxia resulted in the loss of elongated mitochondria (Figure 2B) such that the average length of ventral cord mitochondria decreased by 40% and total number decreased by 30% (Figure 2J,K). Within minutes of reoxygenation, we observed a reversal towards longer mitochondria, and within a few hours mean mitochondrial length and number were restored to pre-anoxic levels (Figure 2C,J,K).

We reasoned that the changes that we observed in mitochondrial length in response to anoxia could be due to changes in mitochondrial dynamics, including fission and fusion. To test this possibility, we examined neuronal mitochondria in mutants that were either defective for fission (e.g., *drp-1* mutants) or fusion (e.g., *eat-3* mutants). Mutants for *drp-1* contained a smaller number of individual mitochondria, and these mitochondria were far more elongated than ever observed in wild type (Figure 2D,J,K), consistent with impaired fission. By contrast, under normoxia mitochondria from mutants for the mitofusin *eat-3* were smaller than wild type and similar in size to those found in anoxia-exposed animals (Figure 2G,J), although more numerous (Figure 2K), consistent with a block in fusion. If the reduction in mitochondrial size during anoxia is a result of changes in fission/fusion dynamics, then mutations in *drp-1* should block the effects of anoxia on mitochondrial length, and mutations in *eat-3* should preclude any additional reduction in size caused by anoxia. We examined *drp-1* mutants following anoxia and during reoxygenation, and we observed that mitochondria underwent bead-like swelling (Figure 2E,F) but did not complete fission into smaller organelles (Figure 2J), suggesting that anoxia triggers the formation of constriction sites that fail to become severed due to the absence of DRP-1. Moreover, mitochondria in *eat-3* mutants showed no change in size following anoxia and reoxygenation compared to wild type (Figure 2G,H,I). Taken together, our results suggest that anoxia can induce changes in mitochondrial size that are dependent on the mitochondrial dynamics machinery.

### The hypoxia response pathway regulates mitochondrial hyperfusion upon anoxia recovery

**Figure 3:**
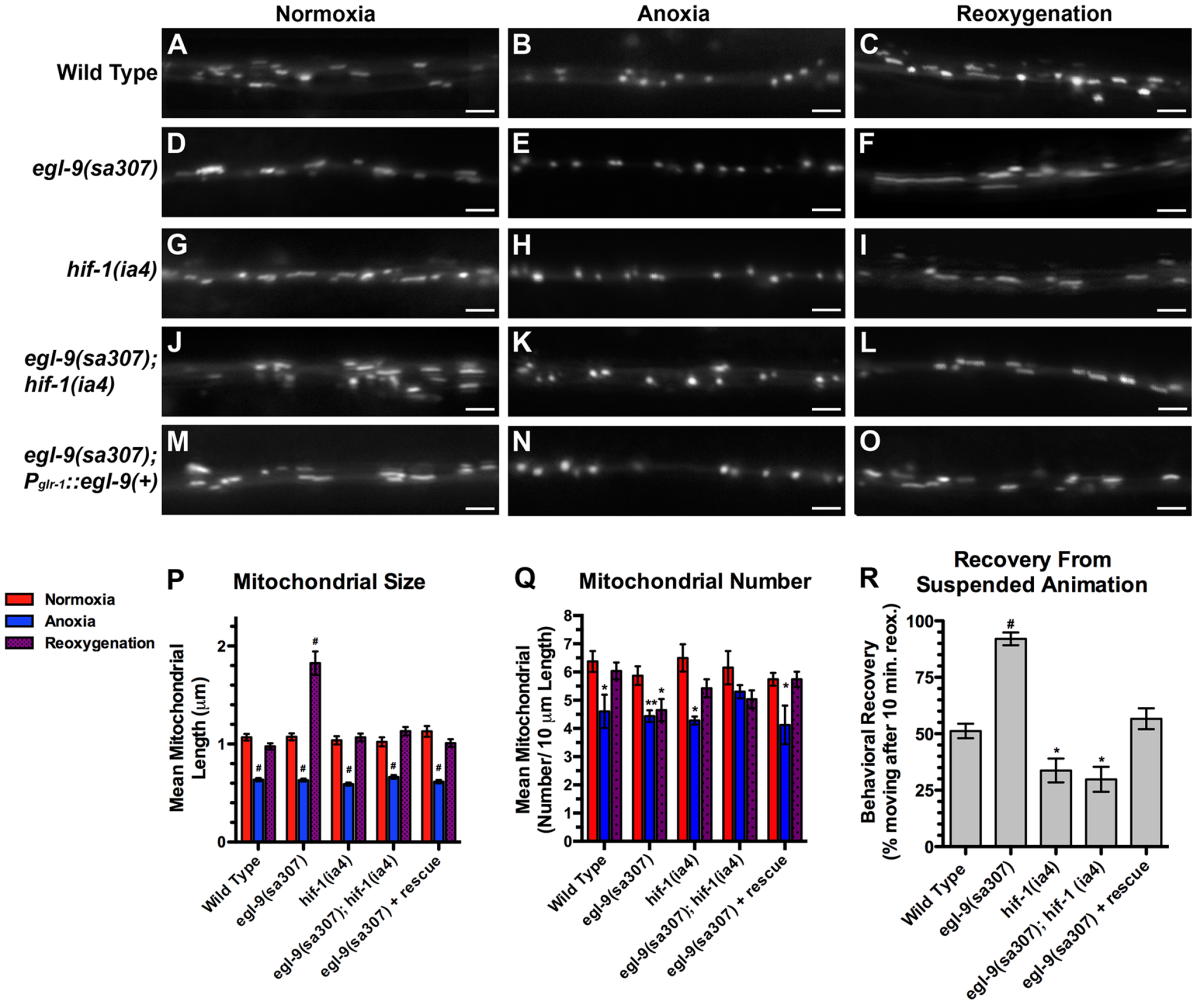
The hypoxia response pathway regulates mitochondrial hyperfusion upon anoxia recovery. The fluorescence of MitoGFP was observed along ventral cord neurites of (A,B,C) wild-type animals, (D,E,F) *egl-9(sa307)* mutants, (G,H,I) *hif-1(ia4)* mutants, (J,K,L) *egl-9(sa307) hif-1(ia4)* double mutants, and (M,N,O) *egl-9* mutants with a transgene expressing the wild-type EGL-9A cDNA from the *glr-1* promoter. Conditions included (A,D,G,J,M) normoxia, (B,E,H,K,N) following 24 hours of anoxia, or (C,F,I,L,O) following 3 hours of reoxygenation post-anoxia. (P,Q) Quantification of the mean (P) length and (Q) number of mitochondria along the ventral cord for the indicated genotypes and conditions. (R) Quantification of behavioral recovery (number of animals moving after 10 minutes of reoxygenation) of animals following 24 hours anoxia. Red bars indicate normoxia, blue bars indicate anoxia, and purple stippled bars indicate reoxygenation. ANOVA followed by Dunnett's multiple comparison to wild type, normoxia (#p<0.001, **p<0.01, *p<0.05). N = 15–35 animals per condition and/or genotype. Error bars indicate SEM. Bar, 5 µm.

One possible mechanism by which neurons might sense anoxia and respond by regulating their mitochondrial dynamics is through the hypoxia response pathway, including its key components: EGL-9 and HIF-1 [25]. We examined MitoGFP in the molecular null loss of function mutants *egl-9(sa307)* and *hif-1(ia4)* [46], [47]. Neuronal mitochondria from both mutants were indistinguishable from those of wild type under both normoxic and anoxic conditions (Figure 3A–H,P,Q), indicating that this pathway is not the mechanism by which neurons sense anoxia and facilitate anoxia-induced fission. Surprisingly, mitochondrial size in *egl-9* mutants was restored in 10 minutes upon reoxygenation post-anoxia compared to an hour in wild type, suggesting a more rapid fusion rate (data not shown). Moreover, the total extent of mitochondrial fusion in *egl-9* mutants upon post-anoxia reoxygenation was excessive, resulting in elongated structures (Figure 3F,P,Q), which we refer to as “hyperfused” mitochondria. Introduction of a wild-type *egl-9* transgene with its expression restricted to the command interneurons by the *glr-1* promoter completely rescued the changes in mitochondrial dynamics following reoxygenation observed in *egl-9* mutants (Figure 3M–O,P,Q), suggesting that EGL-9 has a cell-autonomous function for regulating mitochondrial dynamics in response to oxygen deprivation stress.

EGL-9 regulates multiple developmental and behavioral processes through its negative regulation of HIF-1 function [26], [27], [48], [49]. If EGL-9 were regulating mitochondria through HIF-1, then elongated mitochondria should not be observed in *egl-9* mutants that also lack *hif-1* activity. We found that mean mitochondrial size in *egl-9 hif-1* double mutants resembled that of wild-type animals (Figure 3J–L,P,Q), indicating that HIF-1 is required for EGL-9 to regulate mitochondria in response to anoxia-reoxygenation. Interestingly, the mitochondrial number of *egl-9 hif-1* double mutants was not restored upon reoxygenation, suggesting that EGL-9 might regulate mitochondrial number independent of HIF-1 (Figure 3Q).

We also found that *egl-9* mutants rapidly emerged from suspended animation upon reoxygenation compared to wild type (Figure 3R), similar to the rapid rate of mitochondrial refusion observed in these mutants. By contrast, *hif-1* mutants showed a slight but significant decrease in their rate of emergence, and *hif-1* mutations blocked the rapid emergence observed in *egl-9* mutants. We obtained a transgene that expresses a wild-type EGL-9 cDNA under the control of a pan-neuronal promoter [29], introduced it into *egl-9* mutants, and found that it completely restored the emergence behavior to the slower rate observed in wild-type animals (Figure 3R). Our data indicate that EGL-9 activity in the nervous system is sufficient to regulate suspended animation behavior following anoxia treatment.

Because *egl-9* mutants show an increase in mitochondrial fusion upon reoxygenation, we reasoned that increased mitochondrial fusion by itself might explain the rapid emergence of *egl-9* mutants from suspended animation. We tested this possibility by examining the rate at which mutants with altered fusion/fission dynamics reemerged from suspended animation. Whereas half of wild-type animals were awake and moving within 10 minutes of reoxygenation following 24 hours of anoxia, less than 10% of *drp-1* mutants and no *eat-3* mutants had emerged from suspended animation at the same time point (data not shown), suggesting that the dynamic nature of mitochondrial morphology per se might be required for animals to adapt and respond to anoxic stress. Alternatively, the complete block of fission or fusion in these mutants might compromise organismal health so as to preclude drawing informative conclusions from stress-induced behavioral assays.

### Mitochondrial hyperfusion requires the canonical mitochondrial fusion machinery

**Figure 4:**
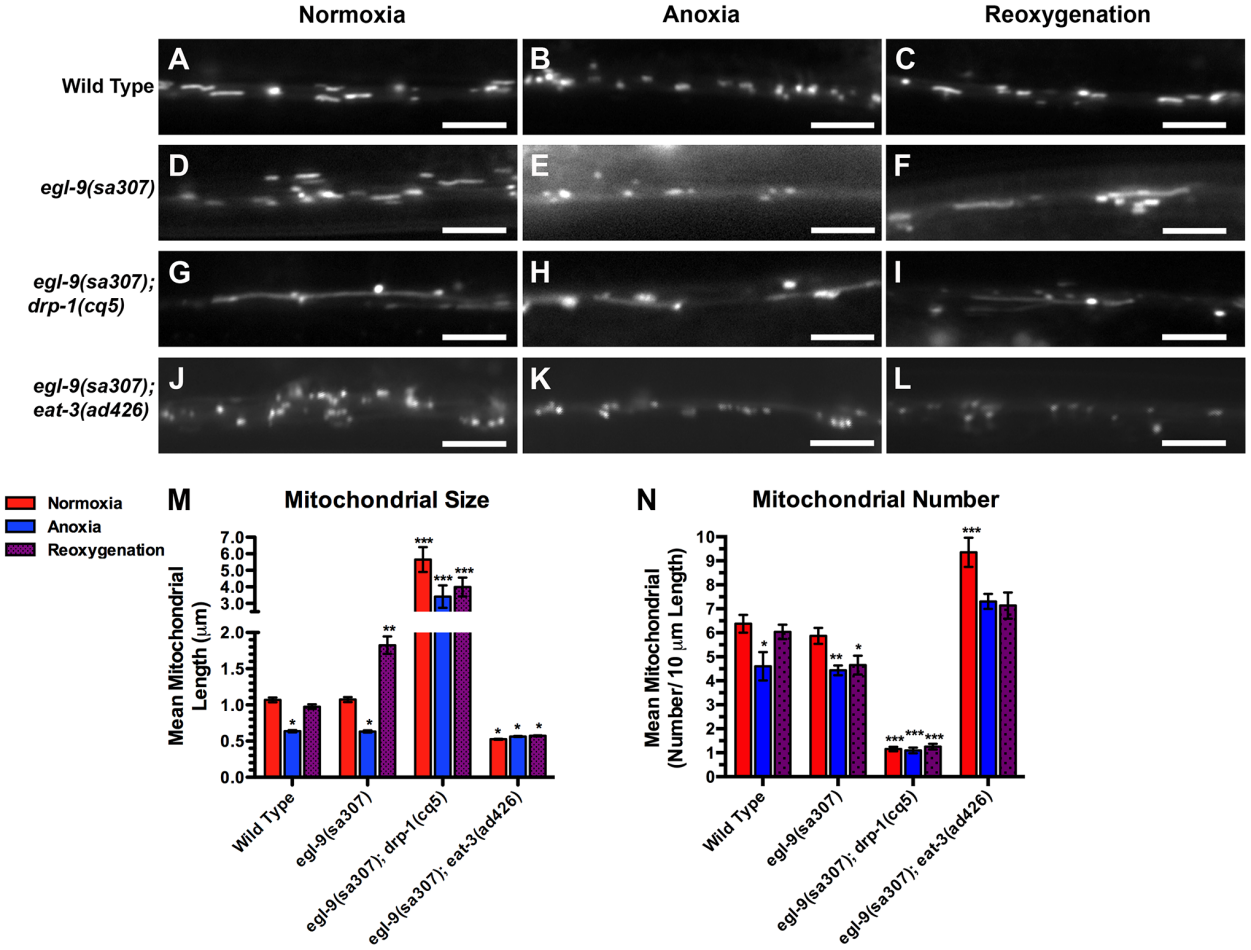
Anoxia-induced mitochondrial hyperfusion requires the canonical mitochondrial fusion machinery. The fluorescence of MitoGFP was observed along ventral cord neurites of (A,B,C) wild-type animals, (D,E,F) *egl-9(sa307)* mutants, (G,H,I) *egl-9(sa307) drp-1(cq5)* double mutants, and (J,K,L) *egl-9(sa307) eat-3(ad426)* double mutants under conditions of (A,D,G,J) normoxia, (B,E,H,K) following 24 hours of anoxia, or (C,F,I,L) following 3 hours of reoxygenation post-anoxia. (M,N) Quantification of the mean (M) length and (N) number of mitochondria along the ventral cord for the indicated genotypes and conditions. Red bars indicate normoxia, blue bars indicate anoxia, and purple stippled bars indicate reoxygenation. ANOVA followed by Dunnett's multiple comparison to wild type, normoxia (***p<0.001, **p<0.01, *p<0.05). N = 15–35 animals per condition and/or genotype. Error bars indicate SEM. Bar, 5 µm.

The elongated, hyperfused mitochondria observed in reoxygenated *egl-9* mutants could be due to altered activity of the canonical mitochondrial fission or fusion machinery. Alternatively, hyperfusion could be a novel form of mitochondrial fusion. To test these possibilities, we examined double mutants between *egl-9* and either *drp-1* or *eat-3*. Mitochondria from *egl-9 eat-3* double mutants were similar in size to those from *eat-3* single mutants, (Figure 4A–L,M,N), suggesting that EAT-3 is required for the mitochondrial hyperfusion observed in *egl-9* mutants upon reoxygenation. In addition, we did not observe any additional increase in mitochondrial size in *egl-9 drp-1* double mutants compared to the single mutants (Figure 4G–I,M,N), suggesting that anoxia-reoxygenation cannot cause additional mitochondrial fusion in the absence of DRP-1. These findings suggest that the hyperfusion observed in *egl-9* mutants during reoxygenation is not likely to be due to a novel fission/fusion mechanism but instead relies on the canonical mitochondrial dynamics machinery.

One simple mechanism by which anoxia might regulate mitochondrial dynamics would be through the enhanced recruitment, turnover, or stabilization of the mitochondrial dynamics machinery during reoxygenation [50], [51], [52]. To test this possibility, we generated a transgene to express an N-terminally tagged DRP-1 protein under the control of the *glr-1* promoter. We introduced this transgene into nematodes and observed that GFP::DRP-1 was localized to punctate structures in neuron cell bodies (data not shown) and along the ventral cord neurites (Figure S1A). We also visualized the *C. elegans* mitofusin EAT-3 by introducing a similar EAT-3::GFP chimeric transgene into nematodes; EAT-3::GFP was localized to puncta, similar to GFP::DRP-1 (Figure S1D). We exposed animals carrying these transgenes to anoxia and reoxygenation; however, we did not observe any significant changes in GFP::DRP-1 or EAT-3::GFP subcellular localization (Figure S1B,C,E,F). We also examined the localization of these proteins in *egl-9* mutants under the same conditions, but did not observe significant changes (data not shown). These results suggest that anoxia and EGL-9 might regulate mitochondrial dynamics by a mechanism other than DRP-1 or mitofusin recruitment.

### Anoxia induces mitochondrial oxidative stress in neurons

**Figure 5:**
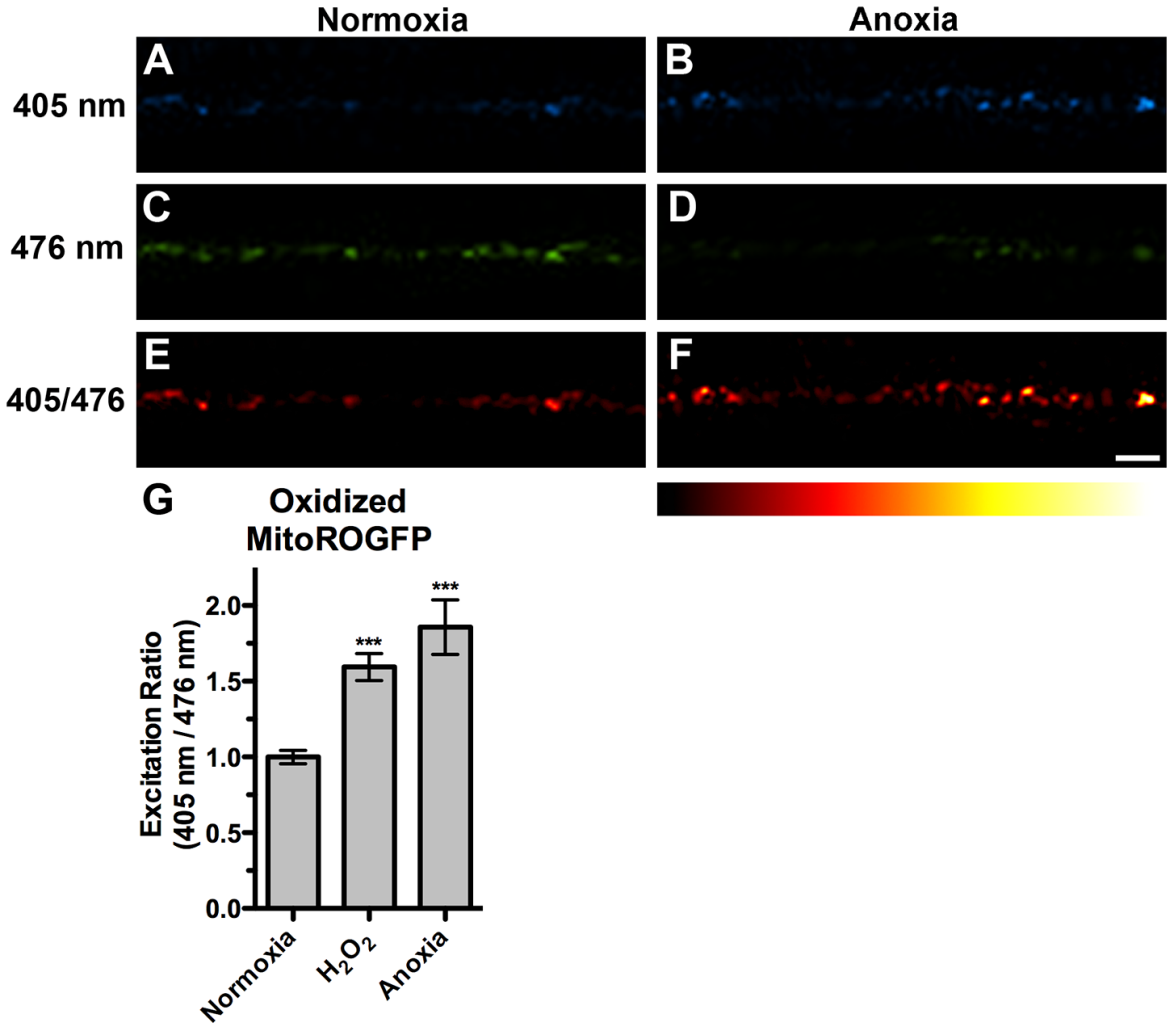
Anoxia induced mitochondrial oxidative stress. The fluorescence of MitoROGFP emitted at a 510(A,B) 405 nm or (C,D) 476 nm light from animals either under (A,C,E) normoxic conditions or (B,D,F) anoxic conditions. (E,F) Ratiometric images were generated from epifluorescence excited by 405 nm light relative to epifluorescence excited by 476 nm light. The ratio has been false colored with the indicated heat map, with high intensity indicative of ROGFP fluorescence in a more oxidative environment. (G) Quantification of mean light ratios evoked by the two excitation wavelengths at individual mitochondria from animals exposed to the given conditions. ANOVA followed by Dunnett's multiple comparison to animals exposed to normoxia (***p<0.001). N = 10–15 animals per condition. Error bars indicate SEM. Bar, 5 µm.

A similar mitochondrial hyperfusion has also been observed in cultured mammalian cells exposed to various stresses, a process termed Stress-Induced Mitochondrial Hyperfusion (SIMH) [20]. In addition, anoxia survival rates in *C. elegans* are influenced by mutations in the insulin/IGF signaling and p38 MAPK pathways, which respond to oxidative stress and ROS [32], [41], [42], [43]. We therefore reasoned that neuronal mitochondria might generate ROS upon anoxic exposure. To test this directly, we generated *odIs111*, a stably integrated transgenic line that expresses MitoROGFP (ROGFP attached to a mitochondrial matrix-directed leader sequence). The fluorescent protein ROGFP (Reduction-Oxidation-sensitive Green Fluorescent Protein) is a modified version of GFP that contains multiple introduced cysteine substitutions, resulting in a protein that upon oxidation demonstrates a rapid, reversible shift in its peak excitation absorbance from 490 nm to 400 nm [53], [54]. We captured confocal images of animals that express MitoROGFP using 405 nm and 476 nm excitation maxima. Under normoxic conditions, MitoROGFP was localized to puncta along ventral cord neurites, similar to MitoGFP, and had a stronger emission when excited by 476 nm compared to 405 nm (Figure 5A,C). To test whether MitoROGFP can report mitochondrial oxidative stress, we exposed nematodes to hydrogen peroxide (H_2_O_2_), a commonly used agent for causing oxidative stress in *C. elegans* [55]. We found that H_2_O_2_ increased the ability of 405 nm light and decreased the ability of 476 nm light to evoke MitoROGFP fluorescence, resulting in 65% increase in the 405/476 excitation ratio (Figure 5G). We also exposed animals expressing MitoROGFP to anoxic conditions and found a shift in emission maxima similar to that of H_2_O_2_ treatment (Figure 5B,D,E–G), suggesting that neuronal mitochondria produce ROS and undergo oxidative stress upon anoxic treatment.

### SKN-1 is required for mitochondrial hyperfusion following anoxia-reoxygenation

**Figure 6:**
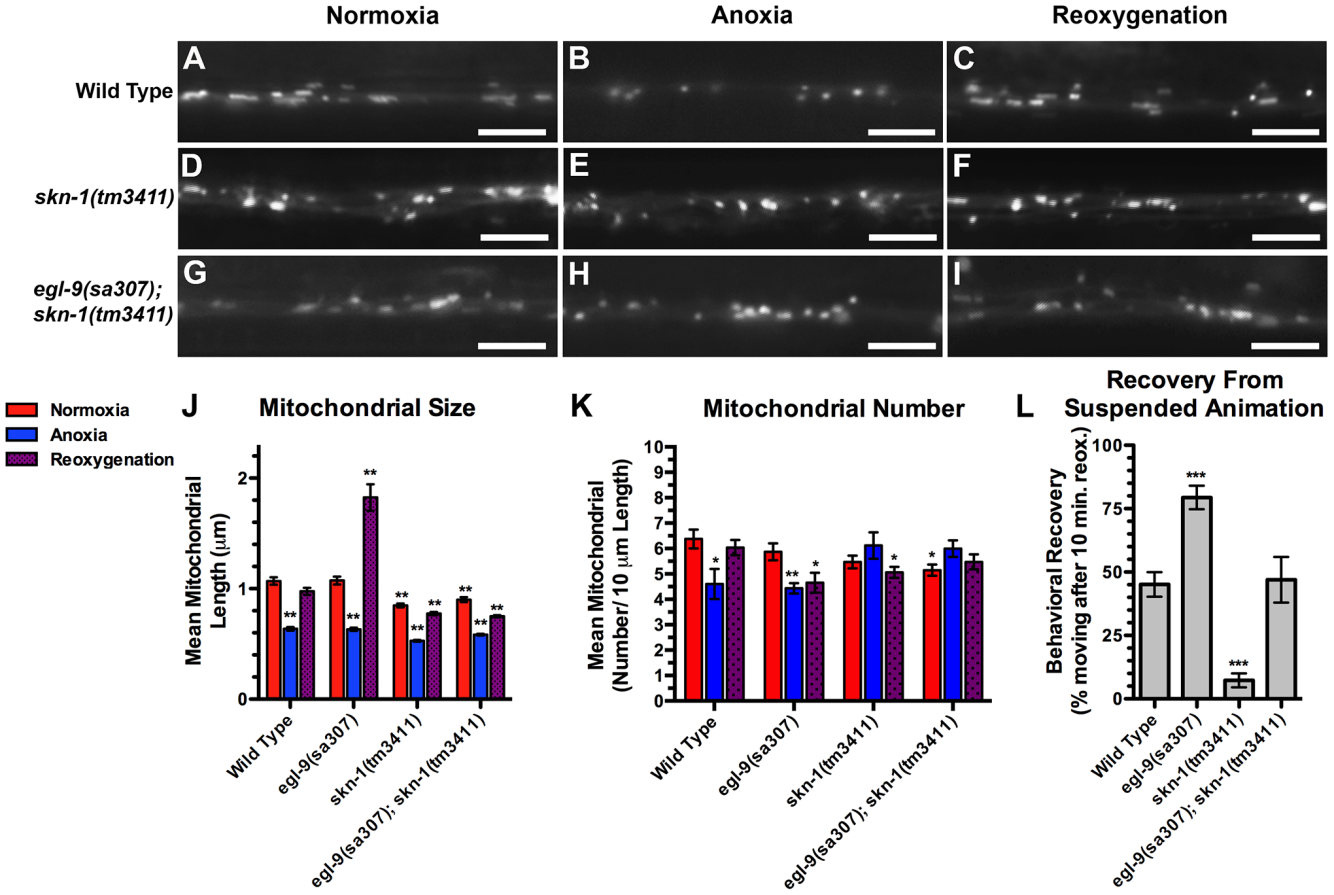
SKN-1 is required for anoxia-induced mitochondrial hyperfusion. The fluorescence of MitoGFP was observed along ventral cord neurites of (A,B,C) wild-type animals, (D,E,F) *skn-1(tm3411)* mutants, and (G,H,I) *egl-9(sa307) skn-1(tm3411)* double mutants under conditions of (A,D,G) normoxia, (B,E,H) following 24 hours of anoxia, or (C,F,I) following 3 hours of reoxygenation post-anoxia. (J,K) Quantification of the mean (J) length and (K) number of mitochondria along the ventral cord for the indicated genotypes and conditions. (L) Quantification of behavioral recovery (number of animals moving after 10 minutes of reoxygenation) of animals following 24 hours anoxia. Red bars indicate normoxia, blue bars indicate anoxia, and purple stippled bars indicate reoxygenation. ANOVA followed by Dunnett's multiple comparison to wild type, normoxia (***p<0.001, **p<0.01, *p<0.05). N = 15–35 animals per condition and/or genotype. Error bars indicate SEM. Bar, 5 µm.

Given that anoxic mitochondria produce ROS, we considered that the oxidative stress response pathway might be required for the corresponding changes in mitochondrial dynamics during anoxia. The transcription factor SKN-1/Nrf is required for the oxidative stress response in *C. elegans* and mammals, and SKN-1/Nrf can associate with mitochondria, where it could directly sense mitochondrial ROS production [55], [56]. Once activated, SKN-1 enters the nucleus and promotes the transcription of phase II detoxifying enzymes and antioxidants to mitigate the harmful effects of oxidative stress [57], [58]. To test whether SKN-1 is required for anoxia-induced mitochondrial hyperfusion, we introduced the MitoGFP transgene into *skn-1* mutants and observed a slight reduction in mitochondrial size in these mutants (Figure 6A,D,J,K). Nevertheless, exposure to anoxia could still trigger an additional reduction in the average mitochondrial size in these mutants, and mitochondrial size was restored after reoxygenation, similar to wild type (Figure 6A–F,J,K). Interestingly, anoxia did not trigger a decrease in mitochondrial number in *skn-1* mutants, suggesting that SKN-1 might be required for changes in mitochondrial number during anoxia. We also examined MitoGFP in *egl-9 skn-1* double mutants and found that the elongated mitochondria observed in *egl-9* mutants were completely absent when *skn-1* mutations were introduced (Figure 6G–I,J,K), indicating that SKN-1 is required for anoxia-induced mitochondrial hyperfusion. In addition, mutations in *skn-1* reduced the accelerated recovery of *egl-9* mutants from suspended animation following anoxia (Figure 6L), suggesting that SKN-1 helps mediate functional recovery from anoxia.

### SKN-1 regulates the expression of prohibitin-like STL-1/SLP-2

To identify potential transcriptional targets of SKN-1 that could explain its role in mitochondrial hyperfusion, we screened through potential SKN-1 binding sites identified in ChIP-seq experiments performed by the modENCODE consortium. A SKN-1 binding region occurs ∼500 bp upstream of the start site of the gene *stl-1* [59]. STL-1 is the lone possible ortholog of mammalian Stomatin-Like Protein 2 (SLP-2), a mitochondrial inner membrane protein required for SIMH in cultured cells [20], with 59% identity and 77% similarity at the protein level to human SLP-2 (STOML2) [60]. Like SLP-2, STL-1 contains a conserved N-terminal mitochondrial leader sequence and a single SPFH domain [61] along its sequence (Figure S2). Conservation between STL-1 and rodent and human SLP-2, as well as a potential Drosophila ortholog, extends along the entire protein (Figure S3).

**Figure 7:**
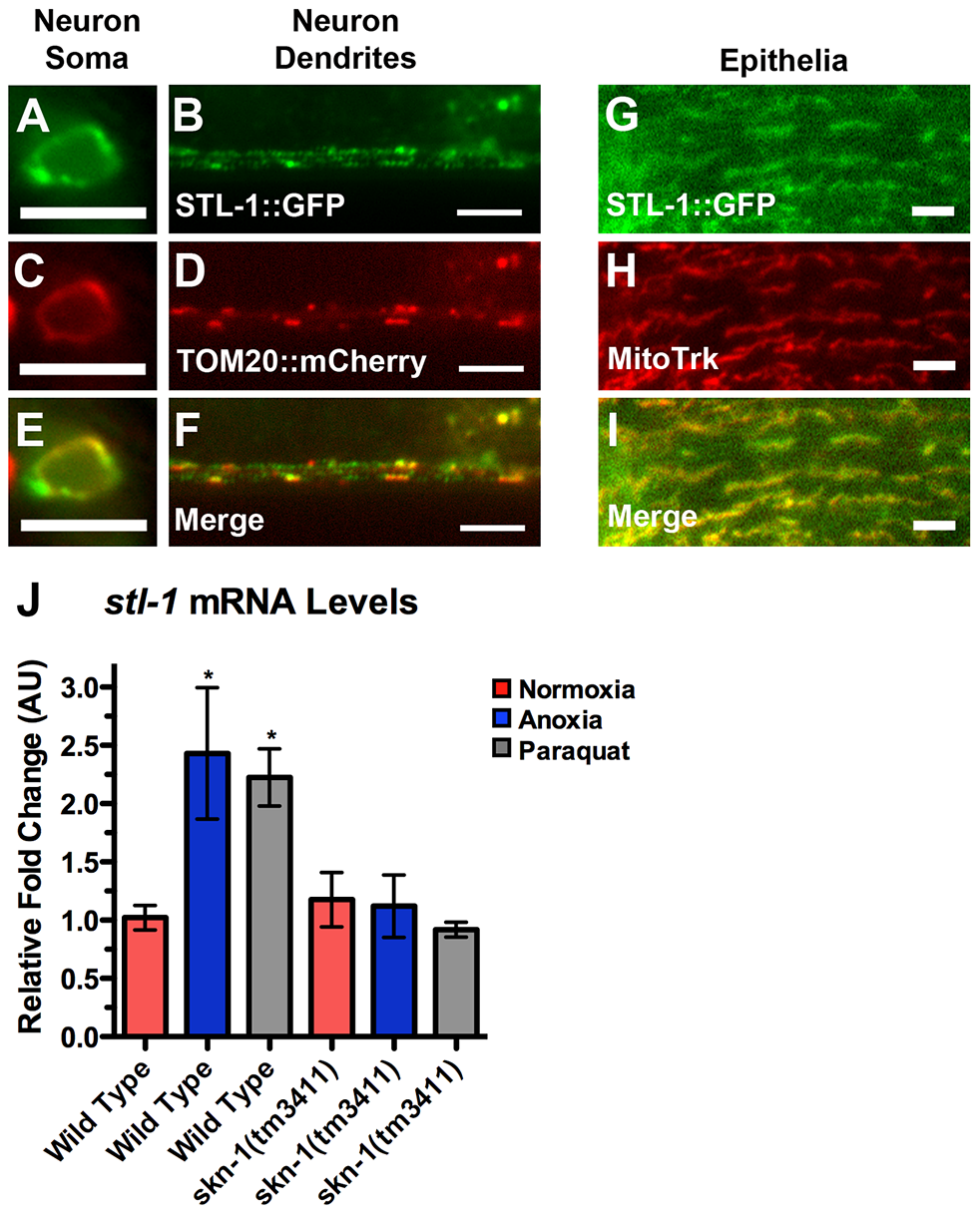
STL-1 resides at mitochondria and is regulated by SKN-1. The fluorescence of (A,B) STL-1::GFP from a *P_glr-1_::STL-1::GFP* transgene, and (C,D) TOM20::mCherry from a *P_glr-1_::TOM20::mCherry* transgene, was observed in (A,C,E) command interneuron cell bodies, including PVC, and (B,D,F) ventral cord neurites of wild-type animals. (E,F) Merged images. The fluorescence of (G) STL-1::GFP from a *P_stl-1_::STL-1::GFP* transgene was observed in the hypodermal epithelia of wild-type animals stained with (H) MitoTracker Red. (I) Merged image. (J) Mean levels of *stl-1* mRNA (relative to wild type) as measured by qRT-PCR in the indicated genotypes and conditions. Red bars indicate normoxia, blue bars indicate anoxia, and gray bars indicate paraquat treatment. Bar, 5 µm.

To confirm that STL-1 is present in neurons, we generated *P_stl-1_::GFP*, a transgene that contains 1 kilobase of sequence upstream from the *stl-1* start codon fused to sequences for GFP (Figure S4A). We introduced *P_stl-1_::GFP* into the nematode germ line to generate transgenic animals and observed GFP expression in all tissues (Figure S4B,C,D). We also generated *P_stl-1_::STL-1::GFP*, which contains the same sequences as in *P_stl-1_::GFP*, as well as the complete open reading frame and introns, resulting in a translational fusion to GFP (Figure S4A); we observed a similar expression pattern using this transgene (Figure 7G and data not shown).

To confirm that STL-1, like SLP-2, is a mitochondrial resident protein, we examined its subcellular localization using two approaches. First, we generated a transgene containing a STL-1::GFP translational chimera under the control of the *glr-1* neuronal promoter. We also generated a separate transgene that contains the mitochondrial resident marker TOM-20::mCherry under the control of the *glr-1* promoter [62]. We generated transgenic lines and observed that both TOM-20::mCherry and STL-1::GFP were localized to elongated structures in a similar fashion to that of MitoGFP in neuron cell bodies and the ventral cord neurites (Figure 7A–D). In addition, we found that TOM-20::mCherry and STL-1::GFP were colocalized (Figure 7E,F). As a second approach, we stained live nematodes expressing *P_stl-1_::STL-1::GFP* with MitoTracker. Whereas MitoTracker cannot penetrate far enough into *C. elegans* tissues to stain neuronal mitochondria, it can label mitochondria in the hypodermis [63]. Hypodermal mitochondria are arranged in elongated networks, and we found that STL-1::GFP was localized to these MitoTracker-decorated networks (Figure 7G,H,I). These results indicate that STL-1 is localized to mitochondria in *C. elegans*, similar to SLP-2 in mammalian cells.

To test whether *stl-1* expression is activated by anoxic exposure and oxidative stress through SKN-1, we first exposed nematodes to the herbicide paraquat, which causes mitochondria to generate superoxide anions [55], [64]. We then isolated total mRNA from these nematodes and measured *stl-1* mRNA levels by qRT-PCR. Paraquat treatment triggered a two-fold increase in *stl-1* mRNA levels in wild type (Figure 7J). We also examined *stl-1* levels in nematodes exposed to anoxia and found a similar increase (Figure 7J). In contrast, we did not observe an increase in *stl-1* mRNA levels in *skn-1* mutants exposed to either paraquat or anoxia (Figure 7J), indicating that SKN-1 is required for *stl-1* regulation by oxidative stress and anoxia.

### STL-1 is required for mitochondrial hyperfusion following anoxia-reoxygenation

**Figure 8:**
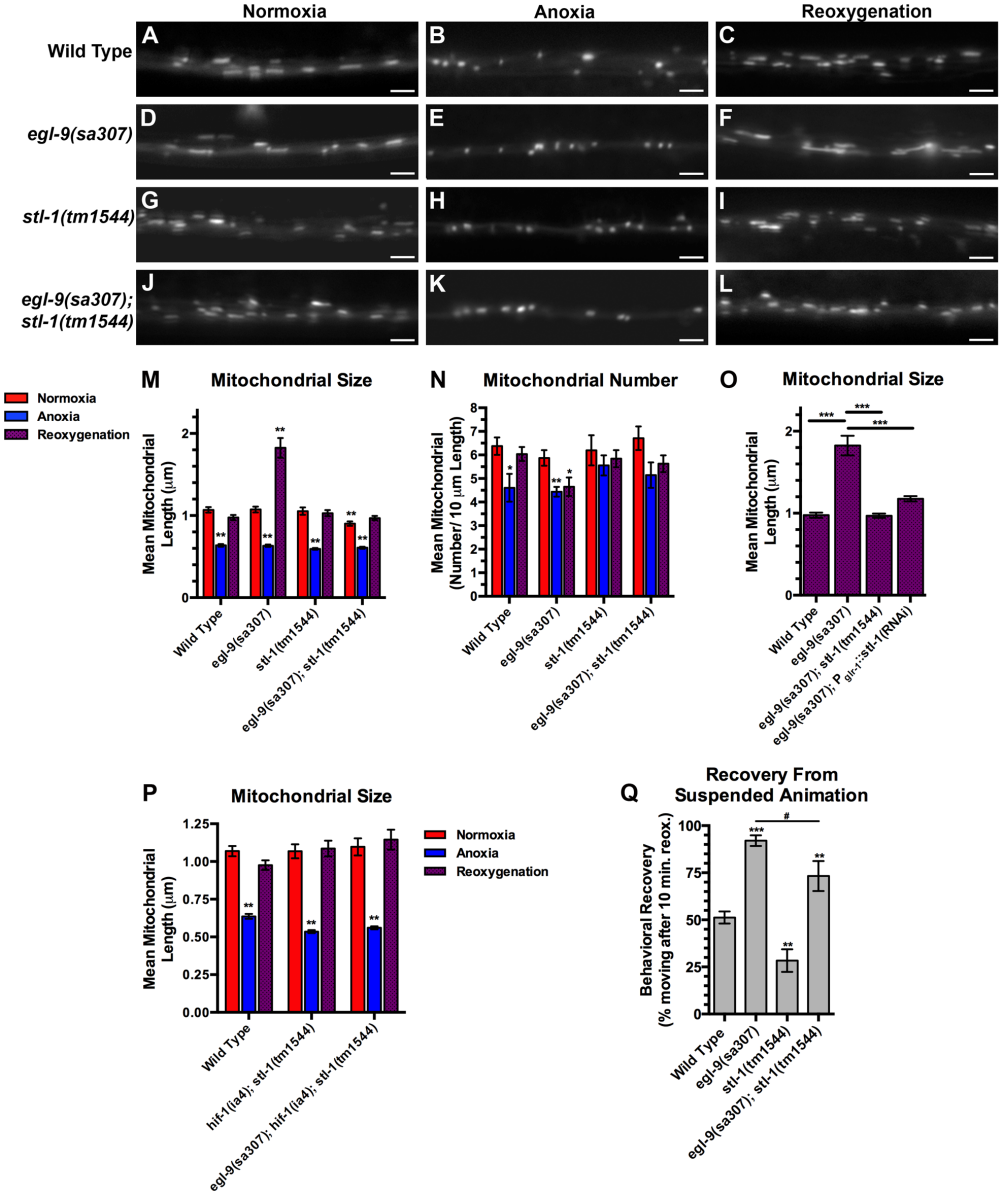
STL-1 is required for anoxia-induced mitochondrial hyperfusion. The fluorescence of MitoGFP was observed along ventral cord neurites of (A,B,C) wild-type animals, (D,E,F) *egl-9(sa307)* mutants, (G,H,I) *stl-1(tm1544)* mutants, and (J,K,L) *egl-9(sa307) stl-1(tm1544)* double mutants under conditions of (A,D,G,J) normoxia, (B,E,H,K) following 24 hours of anoxia, or (C,F,I,L) following 3 hours of reoxygenation post-anoxia. (M–P) Quantification of the mean (M,O,P) length and (N) number of mitochondria along the ventral cord for the indicated genotypes and conditions. (Q) Quantification of behavioral recovery (number of animals moving after 10 minutes of reoxygenation) of animals following 24 hours anoxia. Red bars indicate normoxia, blue bars indicate anoxia, and purple stippled bars indicate reoxygenation. ANOVA followed by Dunnett's multiple comparison to wild type, normoxia (***p<0.001), or by Bonferoni's multiple comparison test for the indicated comparison (#p<0.01). N = 15–35 animals per condition and/or genotype. Error bars indicate SEM. Bar, 5 µm.

Given its similarity to SLP-2, we reasoned that STL-1 is required for mitochondrial hyperfusion following anoxia. We obtained *stl-1(tm1544)*, a mutant that contains a deletion removing half of the *stl-1* gene, including most of the SPFH domain, and results in a frameshift and a premature stop eleven codons following the deletion (Figure S2, S4). Mutants for *stl-1* had no obvious defects in viability, fertility, development, movement, or gross health under standard laboratory conditions (data not shown). We introduced our *P_glr-1_::MitoGFP* transgene into *stl-1* mutants and found that there were normal numbers and sizes of mitochondria in these mutants compared to wild type (Figure 8A–G,M,N). We exposed *stl-1* mutants to anoxia followed by reoxygenation and observed the same changes in mitochondria morphology in these mutants that we observed in wild type (Figure 8H,I,M,N). We generated double mutants between *stl-1* and *egl-9* to test whether STL-1 is required for the hyperfusion that occurs in anoxia-treated *egl-9* mutants. We found that mitochondrial shape and size in reoxygenated *egl-9* mutants was restored to wild-type levels when STL-1 activity was removed (Figure 8L,M), indicating that STL-1 is required for the anoxia-induced hyperfusion observed in these mutants. Mutations in *stl-1* did not restore mitochondrial number in reoxygenated *egl-9* mutants, suggesting that EGL-9 regulates mitochondrial number independently from STL-1 (Figure 8N). Mutations in *hif-1* and *stl-1* when combined did not have an additive effect in suppressing hyperfusion in *egl-9* mutants (Figure 8P). Interestingly, mutations in *stl-1* partially reduced the accelerated recovery of *egl-9* mutants from suspended animation following anoxia, suggesting that STL-1-mediated hyperfusion of mitochondria helps mediate functional recovery from anoxia (Figure 8Q).

If STL-1 is directly mediating hyperfusion in neuronal mitochondria, then it should be required in the same cells as the mitochondria that we are observing. To test this possibility, we generated a transgene containing an inverted repeat of the *stl-1* gene under the control of the *glr-1* promoter to knock down *stl-1* levels solely in the command interneurons by heritable double-stranded RNA interference [65]. We introduced this *P_glr-1_::stl-1(RNAi)* transgene into *egl-9* mutants and found that it had no effect on mitochondrial morphology under normoxia or anoxia conditions. However, the hyperfusion typically observed following anoxia was blocked in *egl-9* with the *P_glr-1_::stl-1(RNAi)* transgene (Figure 8O), suggesting that STL-1 functions cell-autonomously to promote hyperfusion.

**Figure 9:**
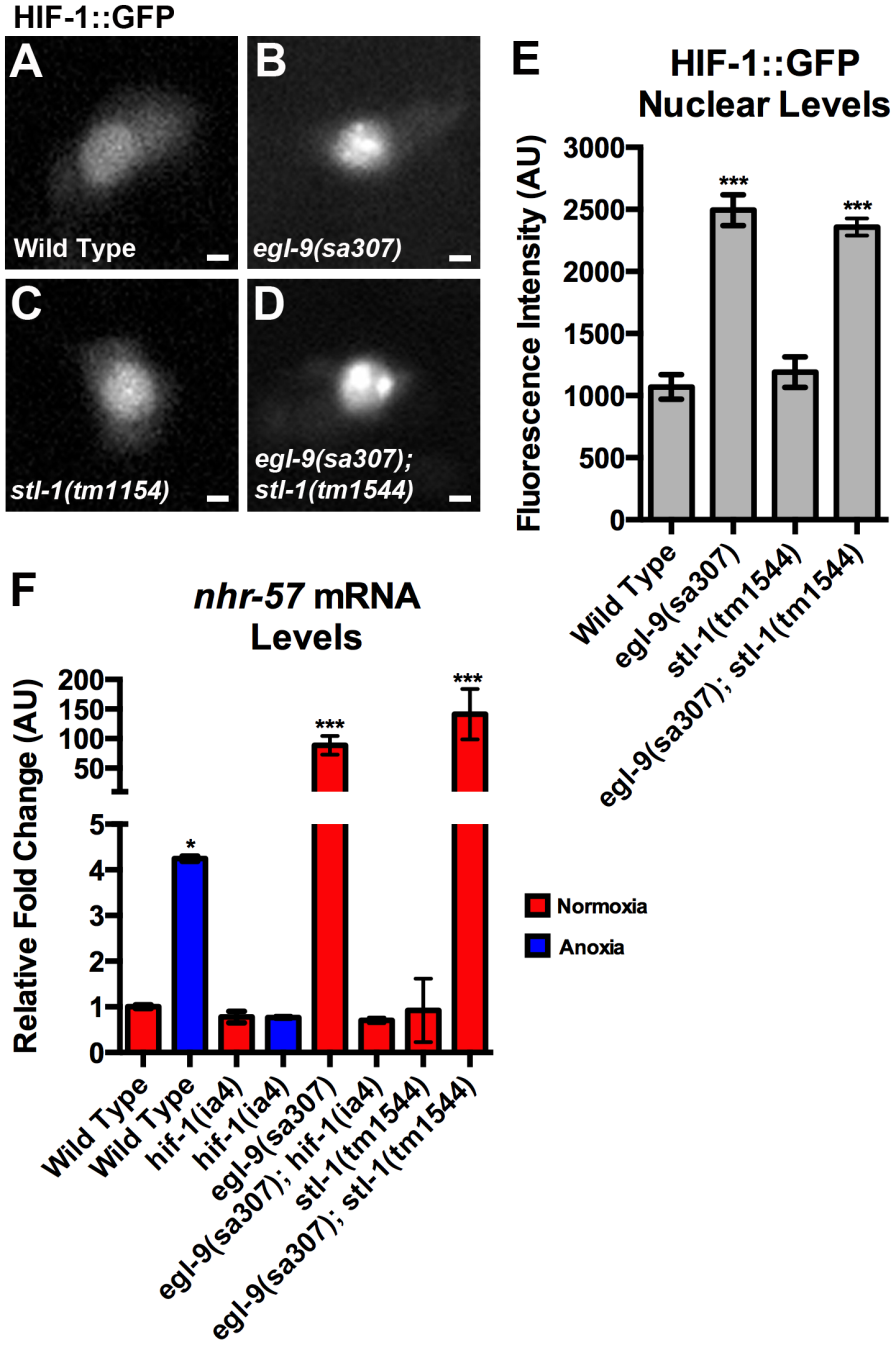
STL-1 is not part of the EGL-9/HIF-1 pathway. (A–D) The fluorescence of HIF-1::GFP from the PVC neuron cell body of (A) wild-type animals, (B) *egl-9(sa307)* mutants, (C) *stl-1(tm1544)* mutants, and (D) *egl-9(sa307) stl-1(tm1544)* double mutants. (E) Quantification of the mean fluorescence level in the nuclei for the indicated genotypes under normoxic conditions. (F) Mean levels of *nhr-57* mRNA (relative to wild type) as measured by qRT-PCR in the indicated genotypes and conditions. Red bars indicate normoxia and blue bars indicate anoxia. ANOVA followed by Dunnett's multiple comparison to wild type, normoxia (*p<0.05, ***p<0.001). N = 3–5 replicates per condition and/or genotype. Error bars indicate SEM. Bar, 1 µm.

One possible explanation for why *stl-1* mutations suppress the effects of *egl-9* mutations is that STL-1 could be part of the hypoxia response pathway, acting to promote HIF-1 function. We think that this is unlikely for several reasons. First, we tested whether HIF-1 levels are regulated by STL-1. EGL-9 negatively regulates HIF-1 by promoting HIF-1 ubiquitin-dependent proteolysis [25]. We generated a transgene containing a HIF-1::GFP chimera under the control of the *glr-1* promoter. We introduced this *P_glr-1_::HIF-1::GFP* transgene into the nematode germ line to generate a transgenic line and observed low-level nuclear HIF-1::GFP accumulation in the neurons of transgenic animals exposed to normal oxygen (Figure 9A). We next crossed this same transgenic line into either *egl-9* mutants or *stl-1* mutants. In *egl-9* mutants, we observed a significant increase in nuclear HIF-1::GFP levels (Figure 9B,E). By contrast, we observed little change in nuclear HIF-1::GFP in *stl-1* mutants (Figure 9C,E). In addition, mutations in *stl-1* did not block the increase in nuclear HIF-1::GFP observed in *egl-9* mutants (Figure 9D,E). Thus, EGL-9 negatively regulates HIF-1 in *C. elegans* neurons, but HIF-1 turnover does not appear to be regulated by STL-1.

We also examined whether STL-1 promotes HIF-1 function by measuring the levels of *nhr-57*, a known transcriptional target of HIF-1 under conditions of hypoxia [66], [67]. We found that anoxia treatment resulted in a four-fold increase in *nhr-57* transcript levels relative to those found in animals under normoxia (Figure 9F). The levels of *nhr-57* were unchanged in normoxic and anoxic *hif-1* mutants relative to normoxic wild type (Figure 9F), indicating that HIF-1 transcriptional activity is increased in response to anoxia, similar to observations of nematodes under hypoxia [67]. As expected, *nhr-57* levels were dramatically elevated in *egl-9* mutants but not in *egl-9 hif-1* double mutants (Figure 9F). By contrast, mutants for *stl-1* contained similar levels of *nhr-57* mRNA compared to wild type. Moreover, *nhr-57* mRNA levels in *egl-9 stl-1* double mutants were unchanged relative to *egl-9* single mutants, suggesting that STL-1 is not required for HIF-1 to regulate its canonical transcriptional targets. Finally, anoxia induced an increase in *stl-1* mRNA levels in *egl-9* and *hif-1* mutants, similar to the wild-type response (data not shown). Taken together, our results suggest that STL-1 functions independently of EGL-9 and the canonical hypoxia response pathway to promote mitochondrial hyperfusion.

## Discussion

**Figure 10:**
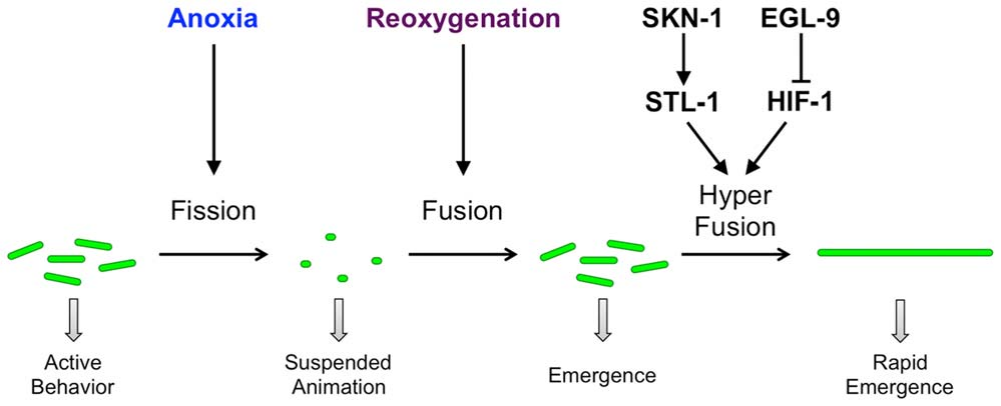
Model for anoxia-induced mitochondrial hyperfusion. Under conditions of normoxia in wild-type neurons, mitochondria undergo a balance of fission and fusion. Exposure to anoxia shifts the balance towards smaller and fewer mitochondria by promoting the canonical fission process. Reoxygenation shifts the balance back towards elongated mitochondria by promoting the canonical fusion process. Depending on the dual activities of the hypoxia response pathway (EGL-9 and HIF-1) and the oxidative stress pathway (SKN-1 and STL-1), reoxygenation can trigger hyperfusion, rapidly resulting in enlarged mitochondria. Mitochondrial dynamics in turn affect the suspended animation behavior of the animal. Hyperfused mitochondria, perhaps through a more efficient generation of ATP, allow neurons to rapidly resume function and rapidly re-emerge from suspended animation. Green ellipses indicate mitochondria distributed along neurites. Arrows indicate stimulatory interactions, whereas T-bars indicate inhibitory interactions.

Here we have shown that oxygen levels regulate mitochondrial dynamics in *C. elegans* neurons, and that the canonical hypoxia response pathway, the oxidative stress response factor SKN-1/Nrf, and the prohibitin-like protein STL-1 can modulate this response (Figure 10). When oxygen levels are sufficiently high, mitochondria exist as a mixture of small and elongated dipolar structures continually undergoing fission and fusion in equilibrium. Under conditions of anoxia, this equilibrium shifts such that smaller mitochondria predominate. In addition, nematodes respond to anoxia by ceasing their movement, entering into suspended animation until conditions improve. Prolonged anoxia results in lethality. However, if the conditions of anoxia are reversed and reoxygenation occurs before the onset of long-term damage, then the animals can emerge from suspended animation and resume their normal development and behavior. Reoxygenation also results in the restoration of the mitochondrial equilibrium to the size distribution that existed prior to the original anoxic stress. Whereas the hypoxia response pathway is not required for the anoxia-induced shift towards smaller mitochondria, it does appear to regulate mitochondrial reconstitution during reoxygenation. In the absence of EGL-9 activity, reoxygenated mitochondria become elongated beyond what is observed prior to anoxia, indicative of hyperfusion. This anoxia-induced hyperfusion is reminiscent of the SIMH that is observed in stressed mammalian cells in culture. Indeed, the mitochondrial hyperfusion that we observed here requires the prohibitin-like STL-1, the *C. elegans* ortholog of SLP-2, a key mediator of SIMH in mammalian cells. Moreover, the expression of STL-1 also requires the transcription factor SKN-1, which is activated by oxidative stress in mitochondria during anoxia. Interestingly, these signaling pathways are also important for modulating the rate at which anoxic animals emerge from suspended animation. We favor a model in which extreme oxygen deprivation activates the hypoxia response pathway and, through the resulting mitochondrial oxidative stress, the transcription factor SKN-1. SKN-1 in turn promotes the expression of the mitochondrial resident STL-1 to augment the rate and extent of mitochondrial refusion upon the restoration of normal oxygen levels. This resulting augmentation results in a more rapid restoration of nervous system function.

*C. elegans*, like many metazoans, encounters zones of low oxygen in its natural environment [23]. Generally, nematodes use specialized sensory neurons and a rapid aerotaxis response pathway to migrate towards areas with an oxygen concentration range of 5–15% [28], [68], [69]. This response is short term (on the order of seconds to minutes), allowing the animal to avoid areas of low oxygen without making dramatic changes in cellular metabolism.

Sometimes the aerotaxis pathways are not sufficient or activated in time to prevent nematodes from entering a hypoxic environment (∼1% oxygen). The conserved hypoxia response pathway becomes activated both to offset the cellular stress of hypoxia in all tissues and to modulate an additional locomotory circuit behavioral response that allows the animals to escape the hypoxic region [25], [30]. The EGL-9 prolyl hydroxylase is the oxygen sensor under these conditions, inhibiting HIF-1. EGL-9 also promotes the activity of glutamate receptors within the command interneurons through a HIF-1-independent mechanism, thereby favoring local foraging behavior. Under hypoxic conditions, EGL-9 becomes inactivated and HIF-1 becomes derepressed, resulting in the upregulation of hypoxia response genes that promote cellular survival under low oxygen conditions; this response includes a rather dramatic shift in strategy for cellular energy production away from oxygen-dependent, mitochondrial-based oxidative phosphorylation and towards anaerobic energy production mechanisms like glycolysis. Hypoxia also results in glutamate receptor activity becoming repressed in the command interneurons, resulting in a switch from local foraging behavior to escape behavior. This is a medium-term response (on the order of minutes to hours) and a sound “escape” strategy, as it allows nematodes to continue to generate sufficient ATP under limited oxygen conditions to propel locomotory behavior so that the animal can flee to areas of higher oxygen concentration.

A more potentially debilitating scenario occurs when nematodes encounter environments of nearly complete oxygen deprivation (anoxia). Under these conditions, maximizing energy production can become a risk rather than an asset, particularly for mitochondria, which generate ROS and release calcium to the cytosol during anoxia. The cellular stress response strategy shifts from maximizing energy production to minimizing energy production and ameliorating oxidative stress. Interestingly, we observed a decrease in both mitochondrial size and number after anoxic exposure, which could be consistent with cells removing potentially dangerous mitochondria during anoxic stress, perhaps by mitophagy, although this remains to be shown. At the organismal level, the stress response strategy also shifts, from one of escape, which requires significant energy production to support locomotion, to one of suspended animation, which minimizes both energy requirements and damage from the associated oxidative stress. This response is long term (on the order of hours to days) and meant to maximize survival during long periods of anoxia until environmental conditions change for the better. Once oxygen is restored, mitochondria become fused again and the original mitochondria number and morphology become restored.

Several signaling pathways modulate survival during anoxia. Mutants lacking the insulin/IGF-like receptor DAF-2 survive for longer periods under anoxia, either because they have elevated expression of genes that offset cellular damage and proteotoxicity, or because they have a reduced metabolic rate [32], [42], [70]. Mutants lacking one of the p38 MAPK pathways also survive anoxia better than do wild-type animals [41]. Preconditioning animals through changes in diet or high temperature growth can also increase survival to subsequent anoxia, perhaps by building up carbohydrate stores in advance of the anoxic period through the activation of AMPK [43].

Our results demonstrate that the EGL-9/HIF-1 hypoxia response pathway is also critical for modulating the response to anoxic stress – a novel role for this pathway. Mutants lacking EGL-9 recover from anoxia-induced suspended animation more rapidly than do wild-type animals; this phenotype can be rescued by restoring EGL-9 solely to the nervous system, suggesting that the suspended animation that is triggered by anoxia might be a regulated function of the nervous system rather than a systemic shutdown of all tissues. Mutants lacking *egl-9* also survive anoxia better than wild-type animals do (data not shown), suggesting that HIF-1, while not required for animals to survive anoxia [44], can promote anoxia survival when its levels are elevated. In this way, *egl-9* mutants provide a model for studying the cellular changes that occur when HIF-1 signaling is elevated due to normal cellular physiology (e.g., in stem cells) or as part of a cellular pathology (e.g., in solid tumors). We speculate that HIF-1 regulates the expression of yet unknown genes involved in mitochondrial dynamics, resulting in fission/fusion machinery that is primed to mediate rapid and extensive changes in mitochondrial morphology and function.

Anoxia results in oxidative stress at mitochondria. Interestingly, a subpopulation of SKN-1 protein is localized at mitochondria, presumably to sense the local oxidation environment [56]. We believe that mitochondrial ROS produced during anoxia activates SKN-1, which in turn promotes mitochondrial hyperfusion and rapid recovery from suspended animation. SKN-1 likely regulates the expression of multiple genes that regulate mitochondrial dynamics and promote recovery from suspended animation following anoxia. One such direct target is STL-1, the *C. elegans* ortholog of the mammalian SLP-2 required for SIMH. STL-1/SLP-2 might mediate hyperfusion by regulating and/or recruiting the fusion/fission machinery [20], [71]. While we did not observe changes in the levels or subcellular localization of machinery components like DRP-1 and EAT-3, it remains possible that anoxia and STL-1 regulate this machinery post-translationally. Alternatively, STL-1/SLP-2 might promote hyperfusion and mitochondrial function by regulating cardiolipin enrichment in mitochondria [72].

For reasons that remain unclear, SIMH hyperfusion results in mitochondria that produce higher ATP levels during stress [20], [73]. Mitochondrial hyperfusion similar to SIMH also occurs in *C. elegans* mutants impaired for electron transport chain activity, further suggesting a relationship between mitochondrial dynamics and the production of cellular energy [74]. Increased energy production from hyperfused mitochondria cannot be explained by fusion alone, as the downregulation of fission proteins leads to less ATP production, most likely due to unbalanced fission/fusion dynamics damaging mitochondria [75], [76], [77]. Perhaps hyperfusion facilitates the restoration of cellular energy by maximizing mitochondrial membrane and cristae, or dilutes damaged mitochondrial contents through intermixing with functional mitochondria. SIMH, the description of which has been restricted to mammalian tissue culture cells, likely has a similar role, and our findings would indicate that SIMH occurs *in vivo*.

## Materials and Methods

### Strains

Animals were grown at 20°C on standard NGM plates seeded with OP50 *E. coli*. Some strains were provided by the *Caenorhabditis* Genetics Center. Strains were backcrossed to our laboratory N2 strain. The following strains were used: *drp-1(cq5), eat-3(ad426), egl-9(sa307), hif-1(ia4), skn-1(tm3411), stl-1(tm1544), odIs70[P_glr-1_::MitoGFP, unc-119(+)], odIs71[P_glr-1_::MitoGFP, unc-119(+)], odEx[P_glr-1_::EGL-9A, ttx-3::rfp], nEx[P_ric-19_::GFP::EGL-9(+), P_unc-25_::mCherry], odIs111[P_glr-1_::MitoROGFP, ttx-3;;rfp], odEx[P_stl-1_::GFP, ttx-3::rfp], odEx[P_stl-1_::STL-1::GFP, ttx-3::rfp], odIs124[P_glr-1_::STL-1::GFP, ttx-3::rfp], odIs125[P_glr-1_::STL-1::GFP, ttx-3::rfp], odIs121[P_glr-1_::TOM-20(N-terminus)::mCherry, ttx-3::rfp], odIs122[P_glr-1_::TOM-20(N-terminus)::mCherry, ttx-3::rfp], odEx[P_glr-1_::HIF-1::GFP], odEx[P_glr-1_::stl-1(RNAi), ttx-3::rfp], odEx[P_glr-1_::GFP::DRP-1, ttx-3::rfp]*, and *odEx[P_glr-1_::EAT-3::GFP, ttx-3::rfp]*.

### Transgenes and germline transformation

The *P_glr-1_::MitoGFP* transgenic plasmid (pOR775) was generated by ligating a PstI/KpnI fragment containing the aspartate aminotransferase mitochondrial leader sequence and GFP sequences from pPD96.32 (A. Fire) into pV6, a vector containing the *glr-1* promoter (a gift from Villu Maricq, University of Utah). The *P_stl-1_::STL-1::GFP* transgenic plasmid (pOR763) was generated by using PCR to amplify the *stl-1* genomic locus plus 1 kb of promoter from fosmid WRM0612bG12, and then by using Gateway to introduce the product into a vector containing a C-terminal GFP. The *P_stl-1_::GFP* transgenic plasmid was generated using a similar approach, but without the *stl-1* coding sequences. Several transgenes were generated by using PCR to amplify cDNA from an OpenBiosystems clone followed by Gateway recombination to introduce the product into a vector containing the *glr-1* promoter and C-terminal GFP; these include *P_glr-1_::STL-1::GFP* (pOR776), *P_glr-1_::TOM-20::mCherry* (pOR769 – contains the first 162 nucleotides of the cDNA), and *P_glr-1_::EAT-3::GFP* (pOR750). The *P_glr-1_::GFP::DRP-1* transgenic plasmid (pOR655) was generated by using PCR to amplify cDNA from an OpenBiosystems clone followed by Gateway recombination to introduce the product into a vector containing the *glr-1* promoter and an N-terminal GFP. The *P_glr-1_::MitoROGFP* transgenic plasmid (pOR809) was generated by chemically synthesizing a ROGFP (Genscript) optimized for *C. elegans* expression based on GFP from pPD96.32. Mitochondrial leader sequence was then added as described above for pOR775. The neuronal-specific EGL-9 rescuing transgene, *nEx[P_ric-19_::GFP::EGL-9(+), P_unc-25_::mCherry]*, was a kind gift from Dengke Ma and Bob Horvitz (MIT).

To knock down the *stl-1* gene, we generated a transgene that synthesized a sense and an antisense mRNA under the control of the *glr-1* promoter [78]. The *stl-1* cDNA was amplified by PCR (forward primer 5′-AAAATGGCGCTAACTAATCGACTTTTAATG-3′ and reverse primer 5′-TCACTTTTTCTTATTGCTCAATGAGTCGTAAAC-3′) and subcloned into pCR8 (Invitrogen) by the TOPO cloning reaction, resulting in sense and antisense donor vectors. The donor cDNAs were then moved into pOR298, a destination vector containing the *glr-1* promoter and *unc-54* 3′UTR, resulting in an *stl-1* sense transgene and an *stl-1* antisense transgene. Plasmids for the sense and antisense transgenes were equally mixed (50 ng/µl each) and introduced into *C. elegans* as described below.

Transgenic strains generated in this study were isolated after microinjecting various plasmids (5–50 ng/µl) described above using the indicated transgenic marker (typically *ttx-3::rfp*, a gift from Oliver Hobert, Columbia University, unless otherwise indicated). All resulting transgenes were introduced into the germ line and followed as extrachromosomal arrays. All nematodes were cultured according to standard approaches.

### Anoxia-reoxygenation

NGM plates seeded with a small lawn of OP50 were used. Approximately 50 N2 (wild-type) animals of L4 stage were picked onto the small amount of food on the plate. Transparent AnaeroPack-Anaero sachets were used to create an anoxic environment, as described above. Methylene blue test strips inside each sachet were used to confirm anaerobic conditions. To normalize each experimental replicate, all genotypes were placed into the same sachet for the times indicated, and control genotypes were carefully evaluated for suspended animation behavior to verify that anoxic conditions were generated. To initiate reoxygenation, plates were recovered from the sachet and returned to standard laboratory conditions for the times indicated.

### Oxidative stress induction

To induce oxidative stress, L4 stage synchronized animals were treated with 200 mM H_2_O_2_ (Fisher-Scientific) in M9 buffer for 1 hr or 150 mM methyl viologen dichloride hydrate (Paraquat, Sigma) in M9 buffer for 1 hr.

### Behavioral video-quantified emergence assay

AnaeroPack-Anaero sachets were used to create an anoxic environment, as described above. Upon reoxygenation, plates were immediately video recorded to observe the animals as they emerged from suspended animation. Videos of 700 pixels×700 pixels were captured at 30 FPS using a Nikon Camera (Mod. No. 352500) and Streampix acquisition software (Version 3.46.0).

### Behavioral simultaneous emergence assay

Strains were synchronized accordingly, and at L4 stage approximately 50 animals were picked to NGM plates seeded with OP50. A wild-type control was included along with the mutant strains, which were outcrossed six times to the control. Following 24 hours of anoxia treatment, the plates were removed and simultaneously quantified for the number of animals in suspended animation versus moving (emerged) at time zero and after 10 minutes.

### Fluorescence microscopy

GFP-, RFP-, and mCherry-tagged fluorescent proteins were visualized in nematodes by mounting larvae on 2% agarose pads with levamisole. AnaeroPack-Anaero sachets were used to create an anoxic environment, as described above. Fluorescent images were observed using a Zeiss Axioplan II. A 100× (N.A. = 1.4) PlanApo objective was used to detect GFP and RFP signals. Imaging was done with an ORCA charge-coupled device (CCD) camera (Hamamatsu, Bridgewater, NJ) using iVision v4.0.11 (Biovision Technologies, Exton, PA) software. Exposure times were chosen to fill the 12-bit dynamic range without saturation.

The quantification of ventral nerve cord fluorescent mitochondria was done using ImageJ [79] to automatically threshold the images and then determine the outlines of fluorescent objects in ventral cord neurites. ImageJ was used to quantify both the shape and the size of all individual fluorescent mitochondria along the ventral cord. Mitochondrial size was measured as the maximum diameter for each outlined fluorescent object. Object number was calculated by counting the average number of puncta per 100 microns of neurite length.

P_glr-1_::HIF::GFP images for the PVC neuron cell bodies were taken using a confocal microscope equipped with the confocal imager (CARV II; BD) and a 40× Plan Neofluar objective, NA 1.3 (Carl Zeiss). Exposure times were chosen to fill the 12-bit dynamic range without saturation. The quantification of HIF-1::GFP fluorescence intensity in the nucleus was conducted using iVision v4.0.11 (BioVision Technologies) software. After background signals were subtracted, total nuclear HIF-1::GFP pixel intensity was divided by the area to get the mean nuclear intensity value.

### Real Time qRT-PCR measurements

Total RNAs were extracted with Trizol (Invitrogen Co., Carlsbad, CA). L4 stage worms (10–20 animals each) were resuspended in 250 µl of Trizol and lysed by one round of freezing (by liquid nitrogen) and thawing (60°C) with subsequent vigorous vortexing in 4°C for 30 min. PCR was performed in an Eco real-time qPCR system (Illumina, San Diego,CA) using iScriptTM One-Step RT-PCR Kit With SYBR Green (Bio-Rad Laboratories Inc., Hercules, CA) in 20 µL reactions with 20 ng of RNA template. We used as forward (5′-AGAAGCGTGGGTTGTAGAAAG-3′) and reverse (5′-TGTAATGGCTCCTTGCTCAG-3′) primers, respectively, for *stl-1*. We used as forward (5′-CGTGATTGCAGACTTGAAAGC-3′) and reverse (5′-GCGTTTGACTTCCATCGTTTG-3′) primers, respectively, for *nhr-57*. We used as forward (5′-ACCATGTACCCAGGAATTGC-3′) and reverse (5′-TGGAAGGTGGAGAGGGAAG-3′) primers, respectively, for *act-1*. Samples were measured two to three times and average values were used for the calculation of relative fold changes. The relative levels of *stl-1* and *nhr-57* mRNA were normalized to the levels of *act-1* mRNA in each preparation. For each experiment, the value for wild type normoxia was set to 1 and other values were normalized accordingly.

### Confocal imaging of MitoROGFP

To measure the MitoROGFP redox transition after anoxia and H_2_O_2_ exposure, images were collected on a Leica SP5 II confocal microscope (Leica Microsystems, Exton, PA) using a 63× (N.A. = 1.4) oil immersion lens. Samples were alternately excited between a 30% 405 nm UV laser and a 30% 476 nm visible laser with a sequential line scanning method. The emission detection was configured for HYD1 photon counting at 508–513 nm. Images were processed using Leica LAS-AF software (version 2.0.0), Image J, and Adobe Photoshop CS3 (Adobe systems). To compare the intensities of each mitochondrion between two excitation wavelengths (405 nm and 476 nm), the same mitochondrial ROIs were chosen to obtain intensity values for both excitation wavelengths. The 405/476 ratios in each experiment were normalized to the values of wild type normoxia.

### MitoTracker staining

Mitotracker (Invitrogen M7512) stock (1 mM in DMSO) was diluted in M9 to a 1∶1000 working solution. Animals carrying the *P_stl-1_::STL-1::GFP* translational reporter were incubated in this solution for one hour at room temperature and subsequently washed, transferred to standard NGM plates, and allowed to recover for 30 minutes in the dark prior to imaging as describe above.

## Supporting Information

Figure S1 Anoxia does not grossly alter the recruitment of mitochondrial dynamics machinery. The fluorescence from either (A–C) GFP::DRP-1 or (D–F) EAT-3::GFP was observed along ventral cord neurites of wild-type nematodes under conditions of (A,D) normoxia, (B,E) following 24 hours of anoxia, or (C,F) following 8 hours of reoxygenation after anoxic exposure. Bar, 5 µm.
(TIF)

Figure S2 Organization of the *stl-1* gene. Genomic sequences of the *stl-1* gene, from the start of translation to the end of translation, are shown, with DNA on the top line and protein sequence listed below. For DNA, capital letters indicate exonic sequences. Numbers are based on nucleotides starting from the ATG. Protein sequence highlighted in gray indicates the predicted mitochondrial localization signal. Protein sequence highlighted in yellow indicates the predicted SPFH domain. Underlined DNA sequences indicate the nucleotides missing in the *tm1544* deletion mutant.
(TIF)

Figure S3 STL-1 is a prohibitin-like ortholog for mitochondrial resident SLP-2. The amino acid alignment of *C. elegans* STL-1 with its putative homologs in humans (STOML2), mice (SLP-2), and *Drosophila* (CG2970).
(TIF)

Figure S4 STL-1 is broadly expressed in most tissues. (A) Schematic of *stl-1* genomic DNA organization and associated transgenes for examining STL-1 expression and subcellular localization. Boxes indicate coding sequences within exons. The arrows indicate the start site of transcription. Brackets indicate regions that encode the indicated protein domains. The green line indicates the region of genomic DNA deleted in the *tm1544* mutant. The green box indicates GFP sequences for the indicated reporter transgenes. (B–D) Fluorescence from *P_stl-1_::GFP* in (B) body wall muscles, (C) intestinal epithelia, and (D) pharynx and head neurons. Bar, 5 µm.
(TIF)
