# Supplementary figures and images for: Anoxia-Reoxygenation Regulates Mitochondrial Dynamics through the Hypoxia Response Pathway, SKN-1/Nrf, and Stomatin-Like Protein STL-1/SLP-2
Source: PLoS Genet. 2013 Dec 26;9(12):e1004063. doi: 10.1371/journal.pgen.1004063 (PMC3873275; doi:10.1371/journal.pgen.1004063)

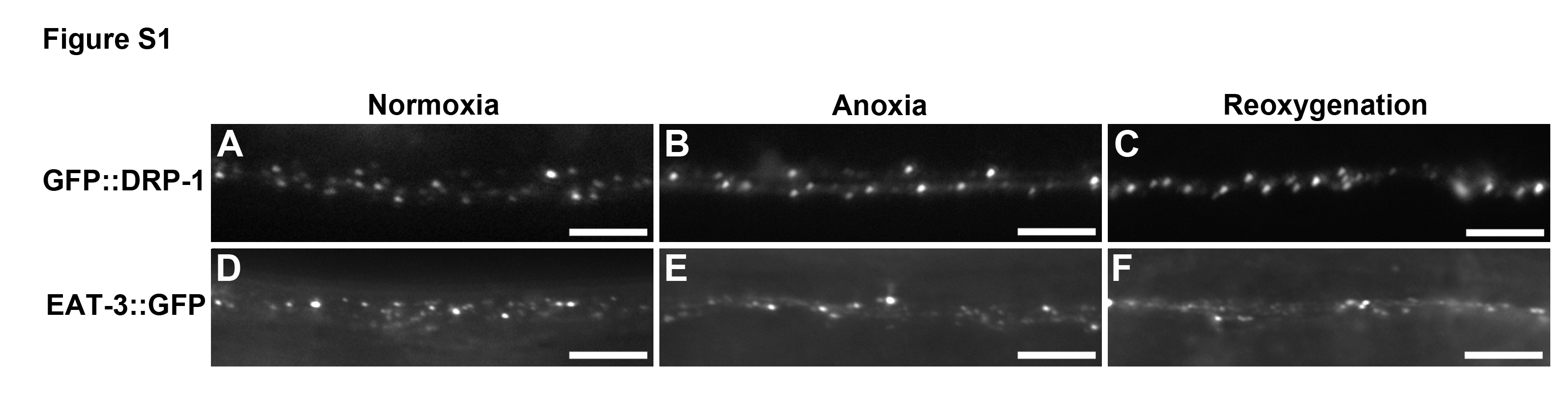

Supplement: Figure S1 — Anoxia does not grossly alter the recruitment of mitochondrial dynamics machinery. The fluorescence from either (A–C) GFP::DRP-1 or (D–F) EAT-3::GFP was observed along ventral cord neurites of wild-type nematodes under conditions of (A,D) normoxia, (B,E) following 24 hours of anoxia, or (C,F) following 8 hours of reoxygenation after anoxic exposure. Bar, 5 µm. (TIF) [file pgen.1004063.s001.tif]

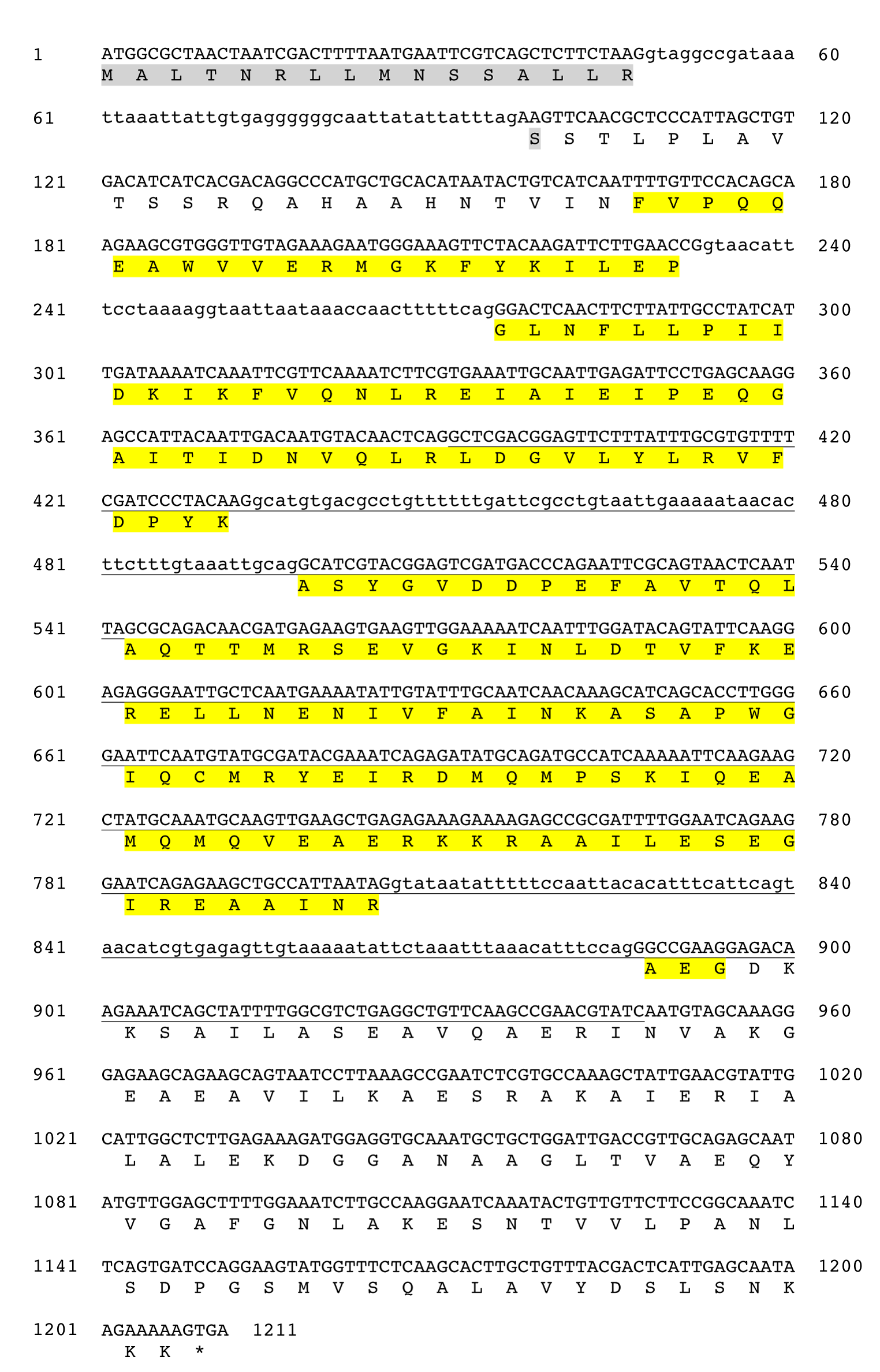

Supplement: Figure S2 — Organization of the stl-1 gene. Genomic sequences of the stl-1 gene, from the start of translation to the end of translation, are shown, with DNA on the top line and protein sequence listed below. For DNA, capital letters indicate exonic sequences. Numbers are based on nucleotides starting from the ATG. Protein sequence highlighted in gray indicates the predicted mitochondrial localization signal. Protein sequence highlighted in yellow indicates the predicted SPFH domain. Underlined DNA sequences indicate the nucleotides missing in the tm1544 deletion mutant. (TIF) [file pgen.1004063.s002.tif]

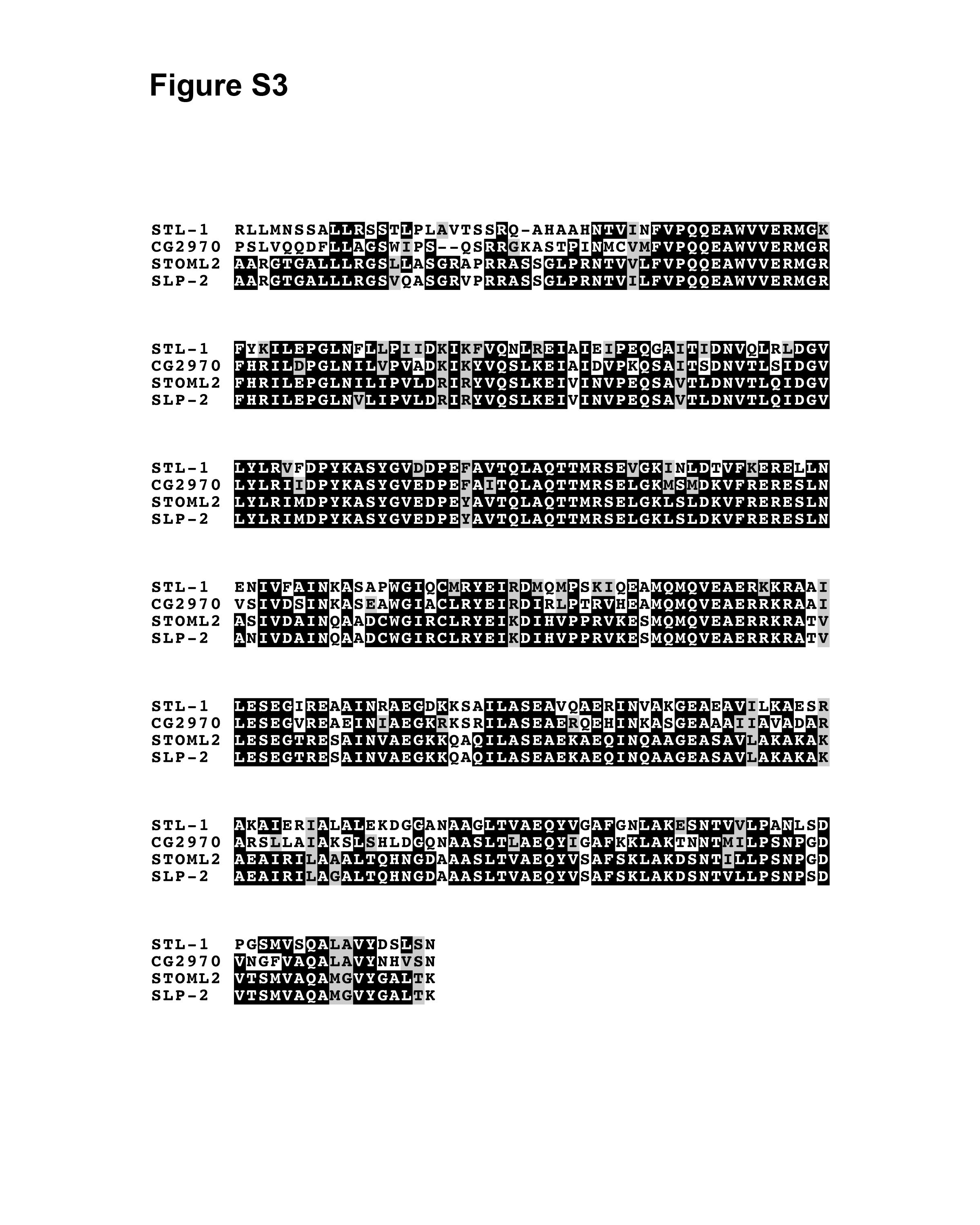

Supplement: Figure S3 — STL-1 is a prohibitin-like ortholog for mitochondrial resident SLP-2. The amino acid alignment of C. elegans STL-1 with its putative homologs in humans (STOML2), mice (SLP-2), and Drosophila (CG2970). (TIF) [file pgen.1004063.s003.tif]

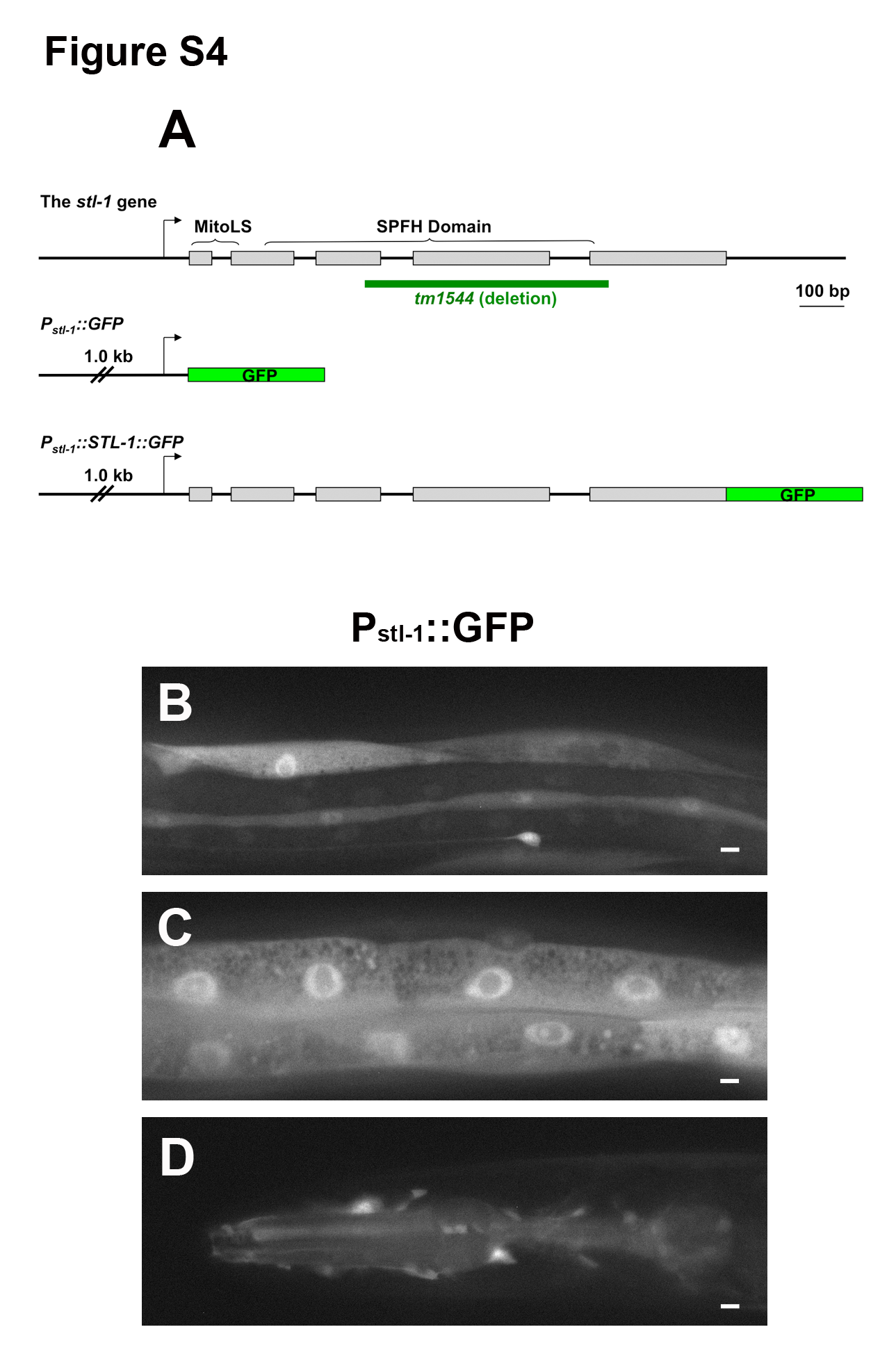

Supplement: Figure S4 — STL-1 is broadly expressed in most tissues. (A) Schematic of stl-1 genomic DNA organization and associated transgenes for examining STL-1 expression and subcellular localization. Boxes indicate coding sequences within exons. The arrows indicate the start site of transcription. Brackets indicate regions that encode the indicated protein domains. The green line indicates the region of genomic DNA deleted in the tm1544 mutant. The green box indicates GFP sequences for the indicated reporter transgenes. (B–D) Fluorescence from Pstl-1::GFP in (B) body wall muscles, (C) intestinal epithelia, and (D) pharynx and head neurons. Bar, 5 µm. (TIF) [file pgen.1004063.s004.tif]
